# Laser-wound stimulated adventitious root formation of *Rosa canina* cuttings involves a complex response at plant hormonal and metabolic level

**DOI:** 10.3389/fpls.2024.1515990

**Published:** 2024-12-16

**Authors:** Raul Javier Morales Orellana, Thomas Rath, Uwe Druege, Yudelsy A. Tandrón Moya, Nicolaus von Wirén, Traud Winkelmann

**Affiliations:** ^1^ Hochschule Osnabrück - University of Applied Sciences, Biosystem Engineering Laboratory (BLab), Osnabrück, Germany; ^2^ Leibniz University Hannover, Institute of Horticultural Production Systems, Section Woody Plant and Propagation Physiology, Hannover, Germany; ^3^ Erfurt Research Centre for Horticultural Crops, University of Applied Sciences Erfurt, Erfurt, Germany; ^4^ Leibniz Institute of Plant Genetics and Crop Plant Research, Department of Physiology and Cell Biology, Gatersleben, Germany

**Keywords:** biochemical signaling, carbohydrates, laser ablation, plant hormones, rooting, rose, wounding

## Abstract

**Introduction:**

The presence of wounds in addition to the excision-induced wounds after severance from the stock plants is known to positively influence adventitious root formation of woody plant cuttings. Previous morphological studies highlighted laser wounding as a technique allowing to precisely control the decisive ablation depth. However, the biochemical processes involved in the response of rooting to the additional wounding remained unexplored.

**Methods:**

The present study analyzed changes in the plant hormone and carbohydrate profiles in response to laser treatments of rose leafy single-node stem cuttings (*Rosa canina* ‘Pfänder’). Concentrations of four groups of plant hormones and of carbohydrates were monitored in three different stem sections of the cutting base during the first eight days after excision of cuttings. In addition, histology was employed to investigate anatomical changes at the basal wound and the laser wounds at the start and the end of the experiment after 40 days.

**Results:**

Laser ablation caused an increase of vascular tissue dimension directly in the laser wound, and increased the quantity and quality of rooting compared to control cuttings. A clear early local rise of jasmonic acid (JA) was detected directly in wounded areas after laser marking, as well as an increase in abscisic acid (ABA) that persisted for the subsequent days. Indole-3-acetic acid (IAA) levels were relatively high on day zero, but decreased thereafter. Interestingly, higher IAA levels were maintained in the stem section below the axillary bud compared with the opposite section. Laser-treated cuttings presented a clear increase in contents of IAA-amino acid conjugates (IAAGlu and IAAsp) and the oxidation product OxIAA. Differences in concentration of these IAA metabolites were related to the position of the laser wound relative to the axillary bud and leaf. Additionally, laser treatments caused gradually increased levels of the cytokinin N6-isopentenyladenine (iP) in laser-treated zones, and of zeatin riboside specifically when the laser wound was placed on the leaf-bud side. Additional laser wounding reduced starch and sucrose levels in all wounded sections at the end of the evaluation period, independently of the wounding location.

**Discussion:**

The results of this study indicate that presence of additional injured tissue triggers a complex biochemical adjustment at the base of the cutting responsible of inducing vascular tissue growth and capable of generating a positive response to adventitious root formation.

## 1 Introduction

Cutting propagation relies on the exploitation of plant plasticity, allowing the formation of adventitious roots (ARs) for the survival of isolated plant parts. The distinct phases of AR formation are described in detail reviewed in Druege et al. (2019). Briefly, the process of AR formation typically begins with the dedifferentiation phase, in cases where the cells from which AR formation starts have not yet acquired root competence. Later, the induction phase takes place, during which the initial cell reprogramming occurs. After the determination of AR founder cells, the initiation phase starts, leading to the formation of new cell clusters into dome-shaped root primordia. Finally, during the expression phase, the root primordia differentiate into a complete root body, followed by root emergence. This capacity has been commonly employed due to its easy practical application, economic feasibility, and reduced time between propagation and commercialization compared to other asexual propagation methods. Various measures have been implemented to enhance rooting percentages and stimulate root abundance, particularly for woody plants due to the limited rooting ability in certain species (reviewed in Diaz-Sala, 2014). One strategy involves additional injured tissue (in addition to the wound generated by excising the cutting) near the cutting base, which stimulates rooting (Hartmann et al., 2002). However, biochemical explanations for the reported rooting stimulation of additional wounds are lacking up to now.

Establishing a precise wounding protocol capable of reaching defined layers of tissue through laser ablation has elucidated how wounding induced changes in rooting percentage and root distribution using rose cuttings (Morales-Orellana et al., 2022). Interestingly, these experiments revealed a positive rooting response linked to the penetration of the laser to the phloem surroundings, particularly when the wound was applied in the form of a strip pattern below the axillary bud. From a histological perspective, an increase in AR formation was related to an evident development of new vascular tissue directly in the laser-wounded areas, which depended on the dimensions of the laser marking pattern applied (Morales-Orellana et al., 2024). These pronounced morphological changes are likely caused by a complex biochemical modulation, in which the interaction of various plant hormones is involved, not only at the cutting base, but also in the additional wounded tissue.

In cuttings without additional wounds, the dynamics of organic metabolites such as carbohydrates and plant hormones have been studied resulting in the development of signaling models (Druege et al., 2019; Li, 2021). These analyses showed that the first hours and days after excision are crucial for proper root induction. It is now well-known, for example, that an early increase in stress hormones such as jasmonic acid and abscisic acid occurs directly in wounded zones, i.e., locally (Lee and Seo, 2022). These local signals elicit a systemic response in the cutting, where auxin re-mobilization from upper parts is particularly important for the induction of ARs and the establishment of a new sink and utilization of carbon and nitrogen in the cutting base (Ahkami et al., 2013; Druege, 2020). Similarly, wounding signaling models in plant tissue have also been developed. Cell monitoring of laser-treated tissues has shown an immediate mechanical response of wounded cells and their surrounding neighbors (Hoermayer et al., 2020). According to these authors, one of the fastest effects of wounding is the instantaneous change in turgor pressure and a local increase of auxin signaling in wound-adjacent cells. Recent reports demonstrated that injured tissue influences processes such as auxin canalization and vascular reconnection (Wulf et al., 2019). Moreover, wound presence leads to changes in tissue restorative rates (Marhava et al., 2019), and the production of auxin-related compounds directly from injured areas (Sheldrake, 2021).

For the first time, this study aimed at investigating the effects on hormonal balance and carbohydrate distribution caused by the additional laser wounds near the base of single-node rose cuttings. The focus of our analyses was on four groups of plant hormones: auxins and its conjugates, jasmonic acid, abscisic acid, and cytokinins, which were quantified during the first eight days after setting the rose cuttings. The results provide new insights into the wound-induced stimulation of AR formation, as well as into the effect of wound position relative to the leaf-bud position, where the leaf constitutes an important carbohydrate source and the adjacent outgrowing bud a potential carbohydrate sink and auxin source (Otiende et al., 2021).

## 2 Materials and methods

### 2.1 Cultivation of mother plants

Mother plants of the rootstock cultivar *Rosa canina* ‘Pfänder’ (clone internal code R02-6) were used in this study. These plants were grown in commercial peat substrate (Steckmedium 304: 20% black peat, 70% white peat, 10% perlite, Klasmann-Deilmann, Germany) fertilized with 100 g/m³ of Radigen (Terraflor, Germany) containing 2.0% Fe, 1.5% Cu, 1.0% Mn, 0.8% Mo, 0.6% B, 0.5% Zn, and 5.0% MgO. Stock plants were maintained at 20°C ± 2°C during the day and 16°C ± 2°C at night in the greenhouse at a relative humidity of 70% to 80%. Plants received additional light from sodium lamps (SON-T 400 W, Philips, The Netherlands) which produced an average light intensity of 630 µmol $\;{\text{m}}^{-2}\;{\text{s}}^{-1}$ (PPFD) for 8 h d^-1^. Plant material was harvested at the end of October in 2022.

### 2.2 Laser pattern design

**Figure 1:**
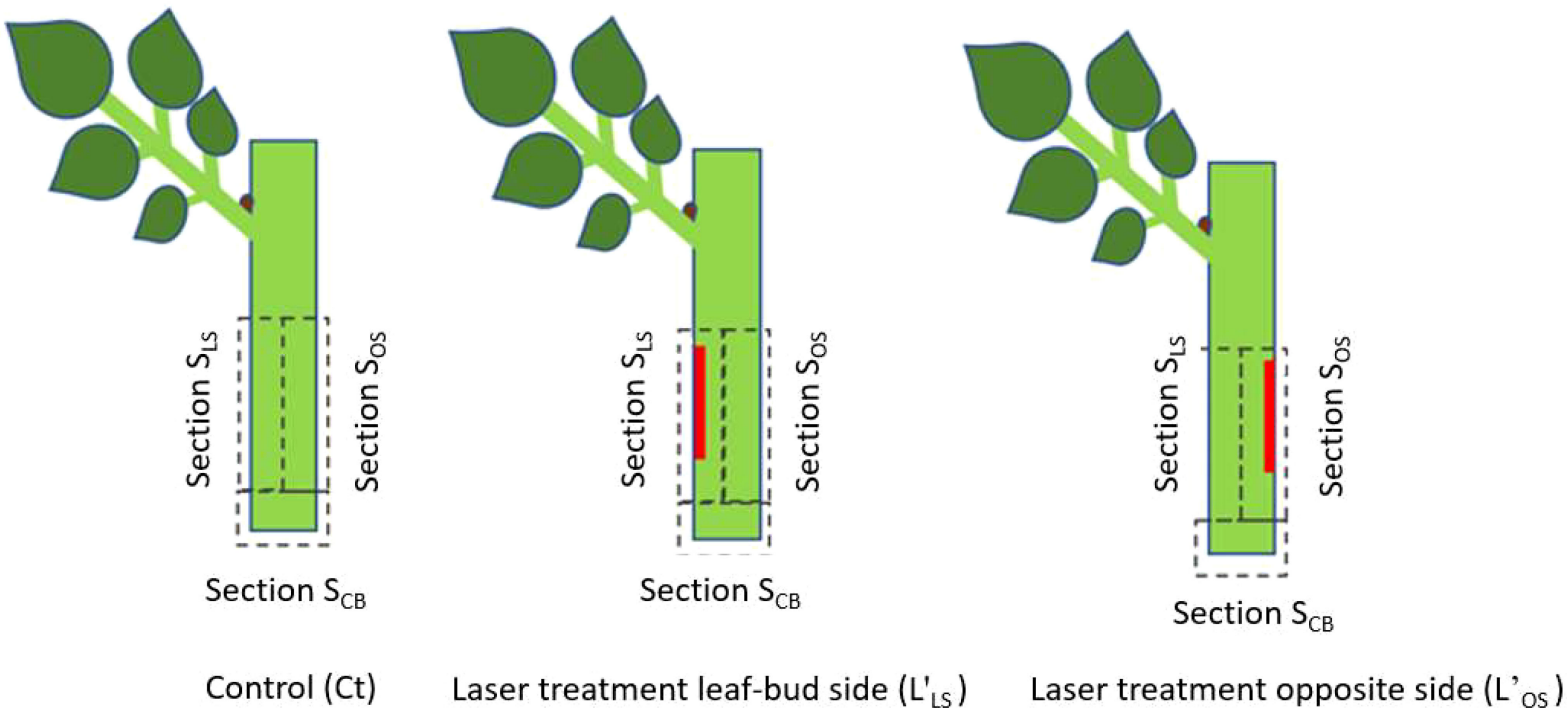
Laser wound treatments (red areas) and sections sampled for biochemical analyses.

The laser marking process was implemented using a continuous wave CO_2_ laser (BRM Pro 600, 80 W power, Netherlands) assisted by a rotary axis (CNC 80 mm Axis Rotary Chuck, LY. Group, China). The laser beam was focused through a 20 mm diameter ZnSe lens (Cloudray, USA), generating a Gaussian beam profile and a laser beam of 240 µm diameter. In order to evaluate the effect of wound position relative to the axillary bud and the leaf, three distinct sections were defined at the bottom part of the cutting: the section above the cutting base on the axillary bud or leaf-bud side (S_LS_); the section above the cutting base opposite to the leaf-bud side (S_OS_); and the section at the cutting base (S_CB_) (**Figure 1**). Two laser treatments were established depending on whether the additional laser wound was located at S_LS_ or S_OS_. Laser treatment on the leaf-bud side is abbreviated in the following with L’_LS_, while laser treatment on the opposite side with L’_OS_. Cuttings without laser treatment (control) were named Ct. The geometry of the laser wound pattern for both treatments was a strip, 10 mm long and 1.0 mm wide, applied around 5 mm above the cutting base. The specific laser energy density required to expose the sclerenchyma, the tissue layer closest to the phloem, was based on the methodology by Morales-Orellana et al. (2022). The average cutting diameter was 2.5 mm ± 0.25 mm.

### 2.3 Cutting propagation conditions

Rooting was carried out under aeroponic conditions, where each aeroponic growth box (measuring 60 cm x 15 cm x 40 cm) was equipped with an independent water pump (WP 25 W, Cadrim, China) containing four Gardena nozzle 360° sprinklers (Germany). The cuttings’ bases were secured using polystyrene rings and received continuous water irrigation through spraying. The pH of the water ranged from 5.5 to 6.5, with an electrical conductivity of 0.18 to 0.24 mS ${\text{cm}}^{-1}$ . No exogenous auxin was applied. In the growth chamber, temperature was maintained between 20°C and 23°C, an average light intensity of 630 µmol m^-2^ s^-1^ (PPFD) was provided by SON-T 400 W sodium lamps (Philips, The Netherlands) for 14 h per day, and relative humidity kept at 80% to 100% through a WHQ 12 head atomizer (DMWD, China) running for 10 min h^-1^.

### 2.4 Cutting treatment, experimental setup and sample collection

The experiment consists of three treatments (**Figure 1**): laser treatment on leaf-bud side (L’_LS_), laser treatment opposite to leaf-bud side (L’_OS_) and cuttings without laser treatment - control (Ct). The experimental setup and timeline are described in detail in **Table 1**. Long shoots were harvested from the stock plants using tree nursery scissors, put in a container with water and transferred to the laboratory, maintaining them wet via water spraying. They were divided into single-node cuttings and stored in distilled water before the laser treatment. After the laser application, cuttings were cultivated in aeroponic cultivation boxes. Tissue samples were taken for biochemical analysis, they were transferred immediately after preparation in dry ice and later stored at -80°C. Sampling for biochemical analyses, rooting evaluation and histological characterization were done in a period of 40 days after treatment (DAT). Selections of mother plants, long shoots, and cuttings were randomized for the three treatments.

**Table 1:** Material collection, cutting treatment and sampling time.

Time	Activity and time specification
8:30 to 9:00 a.m.	**Harvesting of 90 long shoots from mother plants:** wounding of donor shoots due to plant isolation
9:00 to 10:00 a.m.	**Cuttings for biochemical analysis at 0 DAT (45 cuttings)**
	9:00 to 9:05 Dividing long shoots into single-node cuttings, each 2 cm (n = 45), i.e.,first wounding of individual cuttings 9:05 to 9:10 Application of laser treatment L’_LS_ (15 cuttings selected)* 9:10 to 9:15 Application of laser treatment L’_OS_ (15 cuttings selected)* 9:15 to 9:30 Partitioning S_LS_, S_OS_ and S_CB_ from 15 remaining cuttings Ct (45 samples)** 9:30 to 9:45 Partitioning S_LS_, S_OS_ and S_CB_ of laser treatment L’_LS_ (45 samples) ** 9:45 to 10:00 Partitioning S_LS_, S_OS_ and S_CB_ of laser treatment L’_OS_ (45 samples) **
		10:00 to 12:00	**Cuttings for rooting in the climate chamber (315 cuttings)** 9 boxes, each box 35 cuttings*** Filled boxes were immediately transferred to the climate chamber
		12:00	**Additional cutting set for histological characterization at 0 DAT (15 cuttings)** Dividing long shoots into single-node cuttings (n = 15) Sampling of the laser treatments (L’_LS_ and L’_OS_) and control (Ct)

*Laser ablation time plus transferring in the aeroponic box ~ 20 seconds per cutting.

**Partitioning in 3 sections was done manually using a scalpel.

***Every box was filled with 35 samples (11 cuttings per treatment) plus two additional with one treatment (L’_LS_, L’_OS_ or Ct), sum = 35 cuttings.

On each next sampling day (1 DAT, 2 DAT, 4 DAT and 8 DAT) 15 cuttings per treatment were collected randomly from the boxes and partitioned in the three sections S_LS_, S_OS_ and S_CB_ (3 cuttings were pooled, resulting in 5 samples per section and treatment or 15 samples per treatment or 45 samples per time point for the biochemical analyses, each comprising at least 70 mg). On sampling day 40 DAT, 45 cuttings per treatment had remained in the 9 boxes for rooting analysis. From these cuttings rooting percentage, absolute number of AR per treatment, number of roots per rooted cutting, average fresh mass per rooted cutting, and the position from which each root emerged was evaluated. The histological characterization at 0 DAT was done with additional 15 cuttings (5 replicates per treatment) and the histological characterization at 40 DAT was done with 12 cuttings (4 replicates per treatment) that had also been evaluated for their rooting response.

### 2.5 Histological analysis

To evaluate the level of initial tissue penetration of the laser wounding, additional control cuttings and laser-treated cuttings were analyzed (5 cuttings per treatment) via histology as described previously (Morales-Orellana et al., 2022; Wamhoff et al., 2024). Moreover, histological characterization was done with the cuttings at 40 DAT (4 cuttings per treatment). Samples comprised of the whole stem base (S_LS_ and S_OS_) and of the basal wounded section (S_CB_) were fixed in AFE solution (76% ethanol, 1.8% formaldehyde, 20% water, and 5% glacial acetic acid) at 4°C for 24 hours. Dehydration involved a series of ethanol solutions (70%, 80%, 90%, and 96%) under vacuum. The samples were studied following the Technovit^®^ 7100 polymerization protocol (Kulzer, Germany). The pre-infiltration phase was carried out with a solution of basic Technovit 7100 and 96% ethanol in a 1:1 ratio under vacuum for 2 h. During the infiltration phase, the samples were left overnight in the basic Technovit 7100 solution with 1 g of hardener I per 100 ml of basic solution. The embedding was then performed in teflon molds (Histoform S, Technovit^®^) after adding 1 ml of hardener II per 15 ml of the infiltration solution. Samples were sectioned using a microtome (Supercut 2065, Leica, Germany) to a thickness of 8 μm. Subsequently, all samples were stained with the FCA solution according to Etzold (New Fuchsine-Chrysoidine-Astra blue: 1000 ml H2O, 0.1 g New Fuchsine, 0.143 g chrysoidin, 1.25 g Astra blue, and 20 ml glacial acetic acid) (REF 11742, Morphisto, Germany) for approximately 4 min and rinsed with a series of ethanol solutions at 30%, 70%, and 30%. Bark penetration among laser treatments was measured in samples from 0 DAT. Additionally, the area of vascular tissue of stem cross sections in presence and absence of the laser wound was quantified in samples at 0 DAT and 40 DAT. Evaluation was done using a light microscope (BX61, Olympus, Japan) with a digital microscope camera (ColorView II, Olympus, Japan) and quantified with Cell* Imaging Software (Olympus, Japan).

### 2.6 Biochemical analyses

The samples were pulverized in a mixer mill (MM 400 Mixer, Retsch, Germany) in 2 ml reaction tubes with two steel beads for 90 s at a frequency of 30 s^-1^. Then, the frozen material was weighed with a precision scale (AB104SA, Mettler Toledo, United States) and divided into two pre-cooled tubes: one with approximately 20 mg for carbohydrate analysis and the second one with around 30 mg for plant hormone analysis. Special care was taken to avoid thawing.

#### 2.6.1 Carbohydrate analysis

Sugars (glucose, fructose, and sucrose) and starch were determined by enzymatic assays as described by Klopotek et al. (2010) and Lohr et al. (2017). Sugars were extracted in a volumetric mixture of 80% ethanol and 20% imidazole buffer (0.1 M, pH 6.9). After centrifugation (15,000 rpm), the supernatant was shaken for 20 min with activated charcoal (100 mg per ml extract) at room temperature to remove interfering substances. After additional centrifugation, aliquots of the supernatant were pipetted into microplates, dried at 37°C overnight and resolved in imidazole buffer (0.1 M, pH 6.9) containing 1.5 g NADH and 0.6 g ATP per liter. Concentrations of glucose, fructose, and sucrose were measured successively in the microplates via NADH-specific extinction at 340 nm after addition of glucose-6-phosphate dehydrogenase (EC 1.1.1.49), hexokinase (EC 2.7.1.1), phosphoglucose isomerase (EC 5.3.1.9), and invertase (EC 3.2.1.26) (Sigma Chemical Company). After washing the pellets with 80% ethanol and water, starch was fragmented in KOH (0.2 N) at 4°C overnight and at 95°C for 2 h, and then digested by amyloglucosidase (EC 3.2.1.3). The starch concentration was determined via the glucose released as described above.

#### 2.6.2 Plant hormone analysis

##### 2.6.2.1 Plant hormone extraction

For plant hormone analyses, grinded plant material was extracted as described in Milyaev et al. (2022), details are included in the **Supplementary Methods S1** in **Supplementary Data Sheet 1**.

##### 2.6.2.2 LC-MS-MS analysis

The plant hormone measurements were carried out as described in Eggert and von Wirén (2017). Five microliters of purified extracts were injected into an Ultra-performance LC system (Acquity) coupled with a Xevo TQ mass spectrometer (Waters, Milford, MA, USA). The sample analytes were separated on an Acquity UPLC^®^ BEH C18 1.7-μm, 2.1 × 100 mm column coupled with a VanGuard pre-column BEH C18 1.7 μm, 2.1 × 5 mm. The column temperature for all methods was set to 40°C. The autosampler temperature was set to 4°C for auxins, ABA, JA, and CKs. LC and MS methods and used chemicals are described in detail in **Supplementary Methods S2** in **Supplementary Data Sheet 1**.

### 2.7 Statistical evaluation of AR formation, histological and biochemical analyses data

All statistical analyses were performed using the software R 3.3.0 (R Core Team, 2021). AR formation was evaluated with the 135 remaining cuttings in a destructive final evaluation at 40 DAT. The determination of AR formation (yes/no), root number per rooted cutting and root fresh mass per rooted cutting were collected in addition to the number of roots classified by their position relative to the leaf-bud location. For rooting percentage or binomial data, generalized linear mixed models were used assuming a binomial distribution. For the root number per rooted cutting, a generalized linear mixed model was used, while the root fresh mass per rooted cutting was analyzed using linear mixed models. Data of root mass and length are presented as mean values with standard error (SE). The best fitting transformation was identified via the *R* package *bestNormalize* (Peterson, 2021). To test for significant differences in mean values between treatments, the nonparametric Kruskal−Wallis test of the *R* package *agricolae* was employed (Mendiburu, 2021) followed by Dunn’s *post hoc* test for pairwise comparisons.

In the histological section, data were subjected to linear model-based analysis, followed by an analysis of variance (ANOVA) of one factor. When significance was observed, mean values were compared using Tukey’s test (*P* ≤ 0.05) with the *R* package *emmeans* (Lenth, 2022). Data of histology is presented as mean values with standard deviation (SD).

For the biochemical analysis, data were subjected to two-factorial analysis of variance (ANOVA) taking treatment and section plus their interaction as factors. Mean values were compared using Tukey's test (Tukey, *p* < 0.05) with the *R* package *emmeans* (Lenth, 2022). This data is expressed as mean values with standard deviation (SD). The dynamics of each compound was presented for stem sections (S_LS_, S_OS_ and S_CB_) and adjusted as a heat map using the *R* package *gplots* (Warnes et al., 2023). ANOVA results are presented as a table below each heat map. Further graphs were created using the *R* package *ggplot2* (Wickham, 2016).

## 3 Results

### 3.1 Histological analyses of cutting bases at 0 DAT and 40 DAT

**Figure 2:**
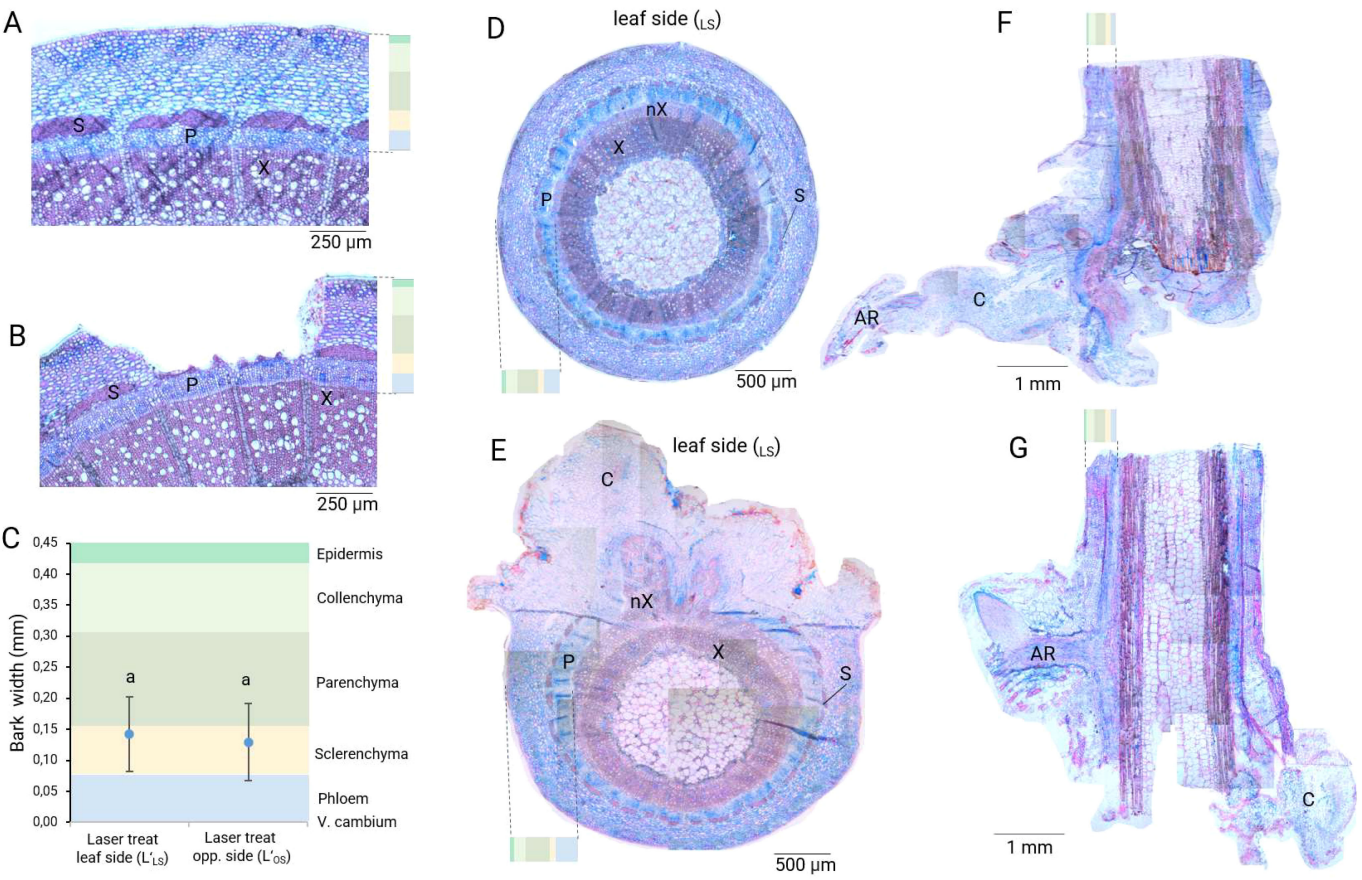
Histological evaluation of the effects of the laser treatments in the different stem tissue layers of rose cuttings (*Rosa canina* ‘Pfänder’). **(A)** Cross section of a control cutting at 0 DAT. **(B)** Cross section of a wounded cutting with the treatment L’_LS_ at 0 DAT. **(C)** Tissue penetration analysis, each color represents the proportion of the respective tissue layers in the bark. Mean values ± SD (n=5). Blue dots indicate the average laser penetration depth. Values with the same letter do not differ significantly (Tukey test, *P* = 0.05). **(D)** Cross section of a control cutting at 40 DAT. **(E)** Cross section of a cutting wounded with the laser treatment L’_LS_ at 40 DAT. **(F)** Adventitious root in the basal wound on a control cutting at 40 DAT. **(G)** Adventitious root in the basal wound on a cutting injured with the laser treatment L’_LS_ at 40 DAT. S, sclerenchyma; X, xylem; nX, new xylem; P, phloem; C, callus; AR, adventitious root.

Histological analyses at 0 DAT showed that both laser treatments reached the sclerenchyma layer and exposed the phloem proximities as intended (**Figures 2A, B**). The whole bark had an average thickness of 0.45 mm (**Figure 2C**). Laser wounding achieved an average depth between 0.25 mm and 0.35 mm for both laser treatments. Therefore, the laser energy applied was able to penetrate the layers of epidermis, collenchyma and parenchyma tissues, until stopping in the fibers of the sclerenchyma, thus, avoiding deeper damage to the vascular tissues (phloem, cambium and xylem). Based on our histological results at 40 DAT, the formation of callus, as well as the radial expansion of phloem and xylem, was necessary for tissue repair and reconnection in the laser wound. A pronounced development of wound-induced xylem and phloem took place directly in the wounded areas produced by the laser pattern (**Figure 2E** versus **Figure 2D**). Basal wound of laser-treated cuttings presented a robust root system directly connected to the sieve elements of the phloem, while control cuttings established AR connections mostly through callus (**Figures 2F, G**).

### 3.2 Rooting response

**Figure 3:**
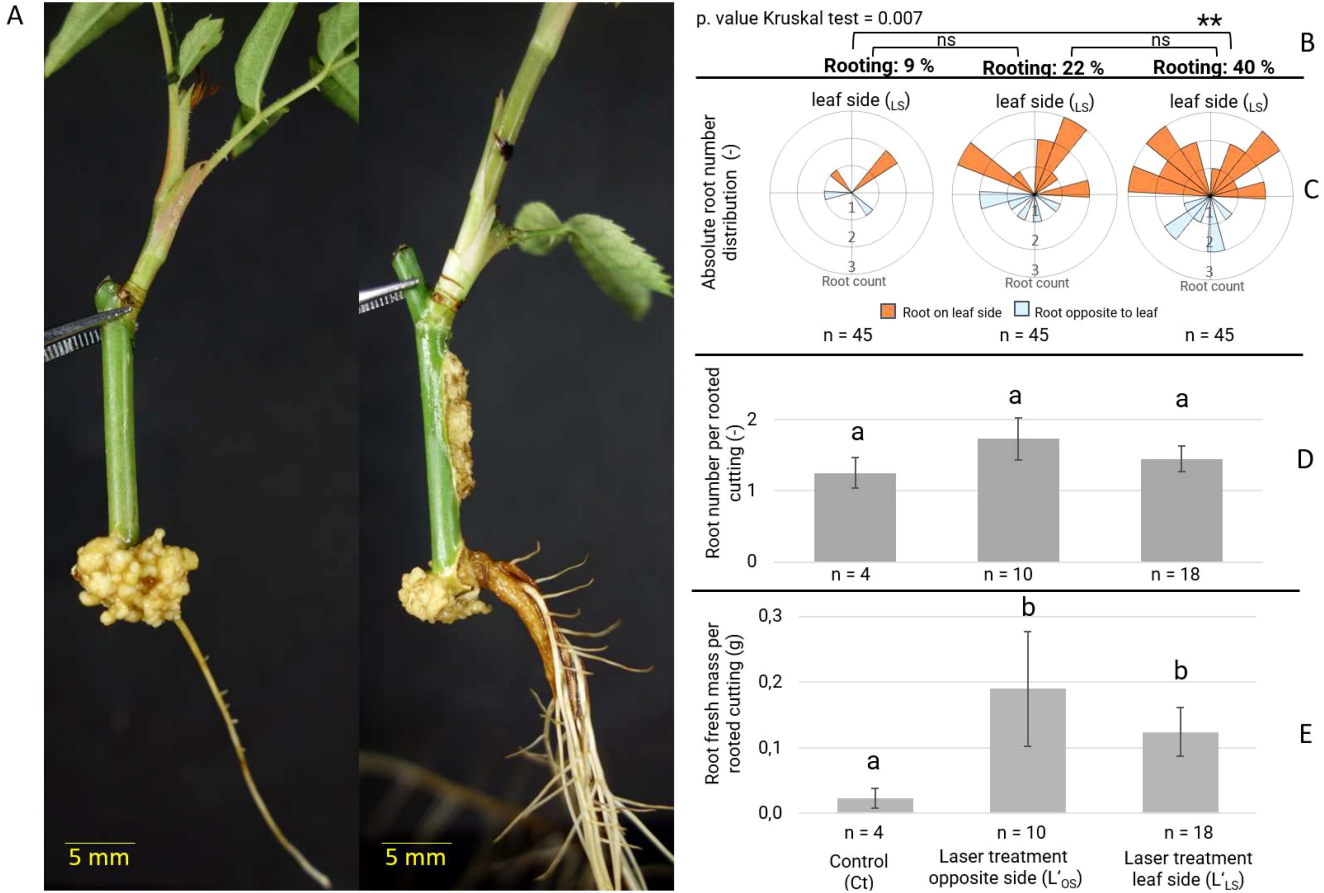
Adventitious root formation of *Rosa canina* ‘Pfänder’ cuttings depending on laser wounding treatments after 40 days of cultivation in aeroponic conditions. **(A)** Comparison between a control (no laser treatment) rooted cutting (left) and a rooted cutting after L’_LS_ laser treatment (right). **(B)** Adventitious root formation expressed in percentage of rooted cuttings indicating significant differences as determined by Dunn’s test (*P* <0.05, n= 45) ** significant at P ≤ 0.01; ns, non-significant. **(C)** Scheme of adventitious root distribution and count of root number per planted cutting (n= 45) classified by their position relative to the leaf-bud side. **(D)** Number of adventitious roots per rooted cutting. **(E)** Root fresh mass per rooted cutting. Data of **(D, E)** are presented as mean values with standard error (SE). Different letters represent significantly different levels between treatments (Tukey test, *P* ≤ 0.05).

The application of both laser treatments resulted in an increase in rooting percentages compared to the control, as well as a significant increase in rooting parameters such as root fresh mass per rooted cutting, but not in the root number per rooted cutting (**Figure 3**). In general, root formation was observed in the basal section S_CB_ of the cuttings in all treatments (**Figure 3A**). At the end of the cultivation period, a significantly higher percentage of 40% of the cuttings was rooted when the laser treatment was applied on the leaf-bud side (L’_LS_) compared to untreated control cuttings. The rooting percentage of cuttings with the laser treatment L’_OS_ was 22%, but this value was not significantly higher than that of the control cuttings with 9% rooting (**Figure 3B**). The root number per planted cutting of 0.4 (18/45) roots for the cuttings of laser treatment L’_OS_ and 0.58 (26/45) for L’_LS_ was about 4 to 5 times higher than that of 0.11 (5/45) roots recorded for control cuttings (**Figure 3C**). The root distribution schemes showed a tendency for the ARs to be positioned preferentially on the leaf-bud side of the cutting, regardless of the treatment. Interestingly, the average number of ARs per rooted cutting was not altered by the presence or absence of additional wounds, being 1-2 roots per cutting regardless of the treatment (**Figure 3D**). However, one of the most notable features of laser-treated cuttings was the vigor of the root system, exhibiting a robust direct connection to the cutting and higher average fresh mass per rooted cutting compared to the control (**Figure 3E**), in which roots were less developed and accompanied by a greater amount of callus. Overall, the laser treatments stimulated rooting and also the position of the wound had an effect on the rooting response.

### 3.3 Plant hormone contents from 0 to 8 DAT

#### 3.3.1 Jasmonic acid and abscisic acid

**Figure 4:**
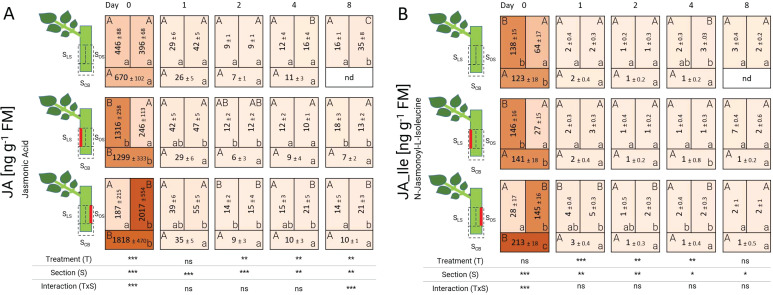
Heat map depicting the distribution of jasmonic acid **(A)** and N-jasmonoyl-L-isoleucine **(B)** analyzed over time and section for the different treatments represented as average ± SD. The results of the ANOVA for the factors treatment and section plus interaction are shown below each heat map. *, **, ***: significant at *P* ≤ 0.05, 0.01, 0.001, respectively; ns, non-significant. Capital letters indicate comparisons of means between treatments per section, while lowercase letters indicate comparisons between sections within each treatment (n=5, Tukey test, *P* ≤ 0.05). nd, no data.

The biochemical analyses showed that jasmonic acid (JA) concentration locally increased at 0 DAT, both at the basal sections S_CB_ of all treatments and in sections where the laser pattern had been applied (**Figure 4**). The concentration of this hormone exceeded 1000 ng g^-1^ FM and it was detected in both laser wounding treatments in the respective wounded section. After 24 h (1 DAT), the concentration of JA decreased drastically in all treatments and sections, but maintaining small local differences in the laser-treated samples. JA concentration reached its lowest value at 2 DAT and kept a low steady level until 8 DAT. N-jasmonoyl-L-isoleucine (JA-Ile) levels presented a very similar pattern to that of JA, but at much lower concentrations: JA-Ile peaked at 0 DAT in all sections with damaged tissue exceeding 100 ng g^-1^ FM. In cuttings of the unwounded controls, the leaf-bud side section S_LS_ also contained a high concentration of 138 ng g^-1^ FM even though the tissue was intact. JA-Ile decreased strongly in all treatments from 1 DAT onwards.

**Figure 5:**
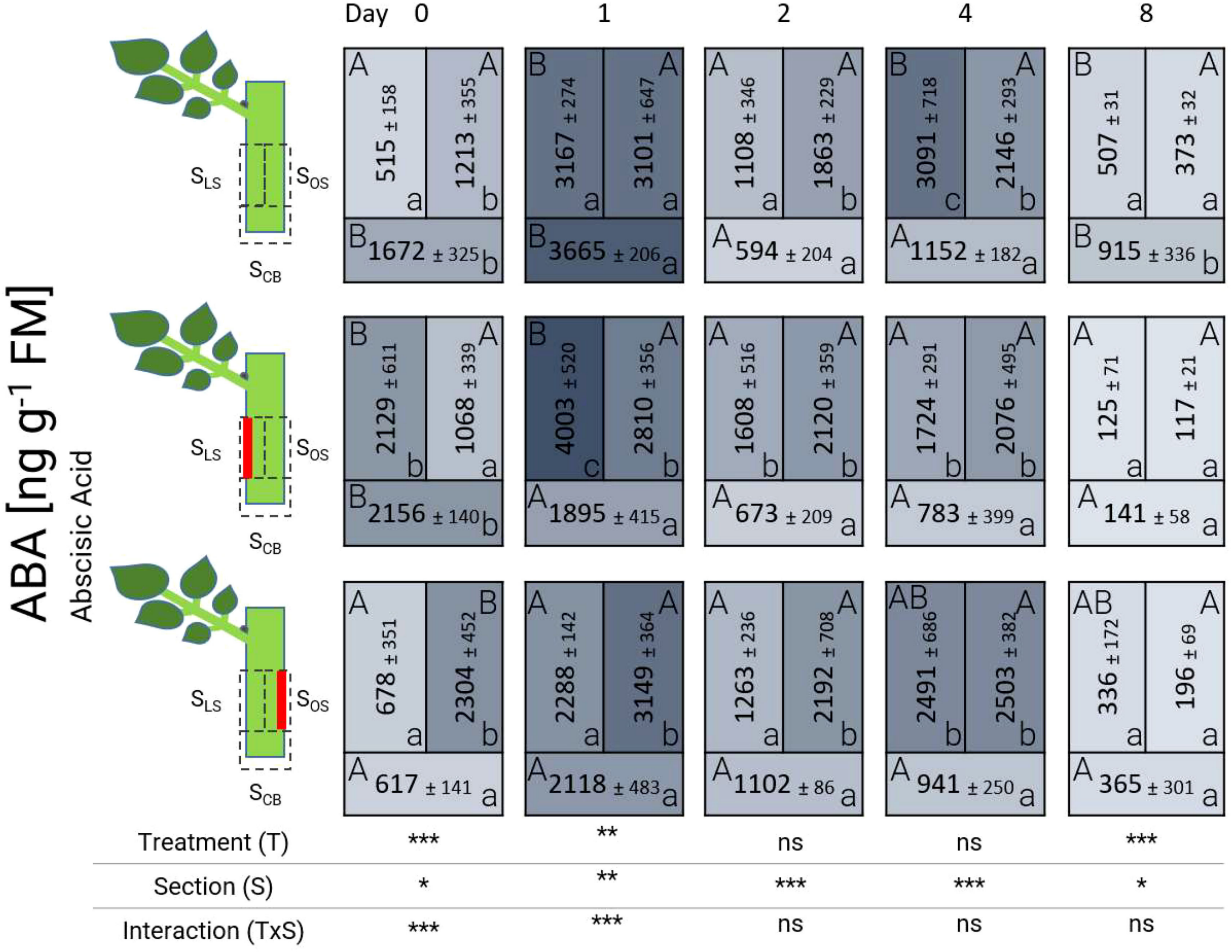
Heat map depicting the distribution of abscisic acid analyzed over time and section for the different treatments represented as average ± SD. The results of the ANOVA for the factors treatment and section plus interaction are shown below each heat map. *, **, ***: significant at *P* ≤ 0.05, 0.01, 0.001, respectively; ns, non-significant. Capital letters indicate comparisons of means between treatments per section, while lowercase letters indicate comparisons between sections within each treatment (n=5, Tukey test, *P* ≤ 0.05).

The concentration of abscisic acid (ABA) displayed fluctuations over time and in different sections (**Figure 5**). Starting from 0 DAT, ABA was highly present in wounded tissue in control and laser-treated cuttings. Just in case of the cutting base (S_CB_) of the laser treatment L’_OS_ at 0 DAT, ABA remained low. After 24 hours (1 DAT), an increase in the concentration of ABA, exceeding concentrations of 2000 ng g^-1^ FM, was noted in all treatments. The content at 1 DAT was particularly high in sections S_LS_ and S_OS_ in the presence of laser-treated tissue as well as in all sections of control cuttings. Between 2 DAT and 4 DAT, the amount of ABA gradually and slowly decreased, reaching average concentrations below 500 ng g^-1^ FM at 8 DAT, with no significant differences between sections (S_LS,_ S_OS,_ or S_CB_) of laser treatments (L’_LS_ and L’_OS_).

#### 3.3.2 Auxin and its derivatives

**Figure 6:**
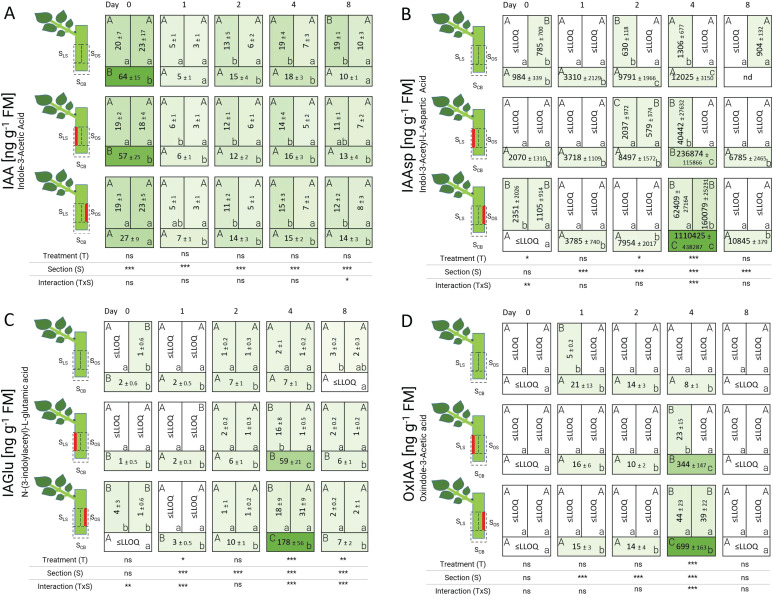
Heat map depicting the distribution of indole-3-acetic acid **(A)**, indole-3-acetyl-L-aspartic acid **(B)**, N-(3-indolylacetyl)-L-glutamic acid **(C)**, and oxindole-3-acetic acid **(D)** analyzed over time and section for the different treatments represented as average ± SD. The results of the ANOVA for the factors treatment and section plus interaction are shown below each heat map. *, **, ***: significant at *P* ≤ 0.05, 0.01, 0.001, respectively; ns, non-significant. Capital letters indicate comparisons of means between treatments per section, while lowercase letters indicate comparisons between sections within each treatment (n=5, Tukey test, *P* ≤ 0.05). nd, no data. ≤LLOQ means Lowest Limit of Quantification.

The concentration of IAA and its derivatives showed two distinct patterns over time (**Figure 6**). First, for the natural auxin, indole-3-acetic acid (IAA), an early high level at the cutting base S_CB_ was recorded in the control and the laser treatment L’_LS_ at 0 DAT, reaching concentrations of over 50 ng g^-1^ FM. In case of laser treatment L’_OS_, IAA concentration in S_CB_ was lower at 0 DAT (27 ng g^-1^ FM) compared to the other two treatments. After 24 hours (1 DAT), the lowest concentrations of IAA were recorded in all sections of all treatments with values below 7 ng g^-1^ FM. The amounts of IAA slightly increased and remained constant from 2 DAT onwards with no significant differences among the treatments and always higher in the leaf-bud side sections compared to the opposite sections independently of additional wounding.

Second, for the IAA-amino acid conjugates indole-3-acetyl-L-aspartic acid (IAAsp) and N-(3-indolylacetyl)-L-glutamic acid (IAGlu), as well as for the inactive form oxindole-3-acetic acid (OxIAA), striking differences between cuttings from laser treatments and the control were detected. In laser-treated cuttings, IAA-amino acid conjugates accumulated in the cutting base S_CB_ over time and reached a pronounced peak with significantly higher levels compared with the control cuttings at 4 DAT. Mostly, at this time point their levels were also higher in the laser-wounded section than in the respective non-wounded section. The highest concentration of all IAA conjugates was noted for IAAsp, in particular at the cutting base of laser-treated cuttings at 4 DAT (**Figure 6B**). The laser treatments also resulted in an increase of IAGlu in S_CB_ of L’_LS_ and L’_OS_, reaching 59 ng g^-1^ FM and 178 ng g^-1^ FM at 4 DAT, respectively (**Figure 6C**). Finally, the product of irreversible oxidation of IAA, OxIAA reached a maximum concentration of 21 ng g^-1^ FM at 1 DAT and thereafter decreased in S_CB_ in control cuttings (**Figure 6D**). In contrast, OxIAA concentrations in S_CB_ of laser treated samples increased until 4 DAT reaching levels higher than 300 ng g^-1^ FM in treatment L’_LS_, and almost 700 ng g^-1^ FM in treatment L’_OS_. Thus, a clear effect of laser treatments on auxin homeostasis in the cutting base at 4 DAT was demonstrated with the accumulation of IAA conjugates and the oxidation product of IAA being stronger in cuttings which were wounded opposite of the leaf and axillary bud (treatment L’_OS_).

#### 3.3.3 Cytokinins

**Figure 7:**
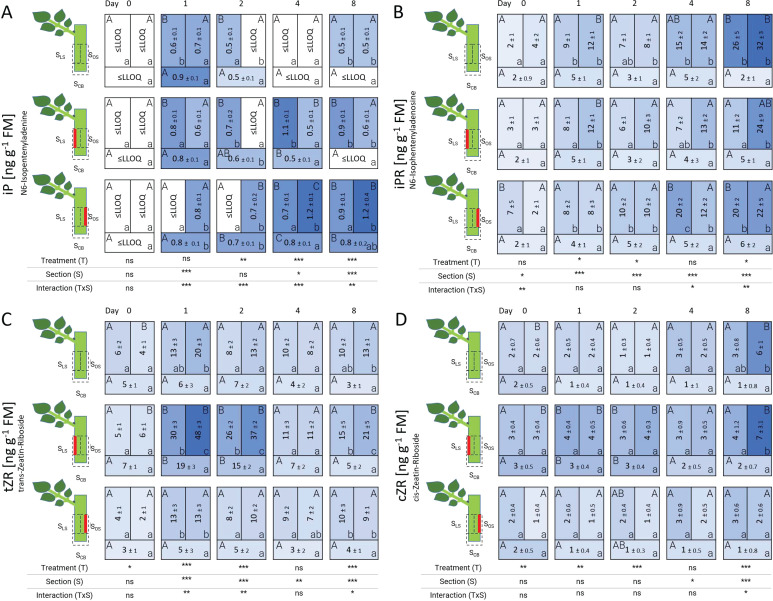
Heat map depicting the distribution of N6-isopentenyladenine **(A)**, N6-isopentenyladenosine **(B)**, trans-zeatin-riboside **(C)**, and cis-zeatin-riboside **(D)** analyzed over time and section for the different treatments represented as average ± SD. The results of the ANOVA for the factors treatment and section plus interaction are shown below each heat map. *, **, ***: significant at *P* ≤ 0.05, 0.01, 0.001, respectively; ns, non-significant. Capital letters indicate comparisons of means between treatments per section, while lowercase letters indicate comparisons between sections within each treatment (n=5, Tukey test, *P* ≤ 0.05).

The analyzed cytokinins differed in their spatial distribution patterns as well as in their dynamics over time (**Figure 7**). The concentration map of N6-isopentenyladenine (iP) illustrates that this cytokinin tended to increase over time specifically in wounded sections although at an overall low level compared to N6-isopentenyladenosine (iPR). The iP concentration did not exceed quantification limits at 0 DAT, whereas at 1 DAT an accumulation of iP was recorded preferentially in the cutting base S_CB_. Between 2 DAT and 4 DAT, iP decreased in all sections of control cuttings to non-quantifiable levels, but remained high for cuttings of both laser treatments. In consequence, additional wounding by laser raised iP levels at 4 DAT in the laser treated sections. Likewise, at 8 DAT, the highest iP concentrations were measured in the laser-treated sections S_LS_ and S_OS_ (**Figure 7A**).

N6-isopentenyladenosine (iPR) started at its lowest levels at 0 DAT with values around 3 ng g^-1^ FM without local concentration differences. From 4 DAT onwards, iPR started to increase, particularly in sections S_LS_ and S_OS_ in all treatments including the control. In control cuttings, the highest levels of iPR were observed at 8 DAT in stem uninjured tissues (S_LS_ and S_OS_), while iPR in the cutting base S_CB_ remained low in all treatments (**Figure 7B**). Laser wounding in the section below the leaf-bud side reduced the iPR level in the same tissue (S_LS_) at 8 DAT.

An increase in trans-zeatin-riboside (tZR) and cis-zeatin-riboside (cZR) was recorded exclusively for cuttings laser-treated on the leaf-bud side (L’_LS_) (**Figures 7C, D**). Similar to iP and iPR, the tZR and cZR content started with low values without differences between sections at 0 DAT. Later, additional wounding of the leaf-bud side increased tZR and cZR levels in all three sections at 1 and 2 DAT. From 4 DAT onwards, zeatin-riboside levels didn’t show major differences among treatments. All cytokinin compounds were lowest in concentration in the section of the cutting base S_CB_.

#### 3.3.4 Carbohydrates

**Figure 8:**
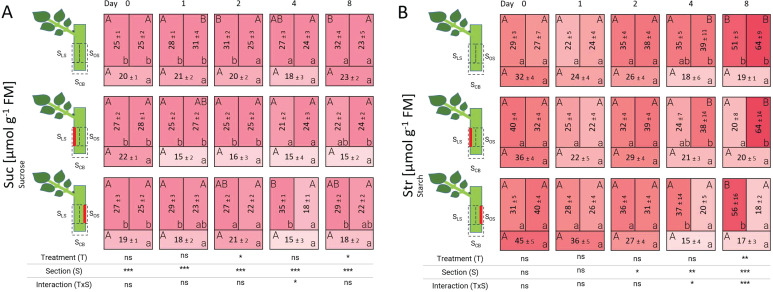
Heat map depicting the distribution of sucrose **(A)** and starch **(B)** analyzed over time and section for the different treatments represented as average ± SD. *, **, ***: significant at *P* ≤ 0.05, 0.01, 0.001, respectively. ns, non-significant. Capital letters indicate comparisons of means between treatments per section, while lowercase letters indicate comparisons between sections within each treatment (n=5, Tukey test, *P* ≤ 0.05).

The distribution of sucrose and starch showed significant differences between treatments especially on the last days of evaluation (**Figure 8**). In case of sucrose, a lower concentration in the basal section S_CB_ was observed compared to the other sections over time for all treatments. From 4 DAT onwards, lower sucrose levels were found in the additionally wounded sections regardless of their position (**Figure 8A**). At 0 DAT and 1 DAT, starch levels did vary neither between sections nor treatments. Starch decreased in the basal section S_CB_ during the evaluation period without any influence of additional wounding of the cutting. However, a local effect of laser wounds was also observed for starch that was even more pronounced than in the case of sucrose. Thus, a decrease in starch occurred in all additionally wounded sections to values below 21 µmol g^-1^ FM at 8 DAT, while in the uninjured sections S_LS_ and S_OS_ the starch concentration reached values higher than 50 µmol g^-1^ FM (**Figure 8B**).

## 4 Discussion

### 4.1 Laser wounding stimulated rooting in the basal wound and the development of new vascular tissue in the additional wounded sections

The rooting results confirmed our previous reports on cuttings of *Rosa canina* ‘Pfänder’, where laser-induced tissue damage led to an increase in the percentages of AR formation, even if no auxin was applied (Morales-Orellana et al., 2024). Therein, we showed that AR stimulation was positively and significantly correlated with an increase in vascular tissue, specifically with phloem enlargement. Furthermore, the first changes in the vasculature of the wounded tissue occurred within the first week, with callus growth being faster than vascular tissue growth. In the present study, the histological results showed that laser wounding increased the development of new vascular tissue directly in laser-treated sections as well (**Figure 2E**; **Supplementary Figure S2**). Similar to our results, studies by Matsuoka et al. (2021) reported an increase of wound-induced cambium and thickening of vascular tissue after wounding in flowering stems of *Arabidopsis.* These authors reported high expression of vascular tissue formation-related genes in these regions. According to Smetana et al. (2019) the regeneration of xylem is necessary to ensure the maintenance of the vascular cambium, making xylem a “stem-cell organizer” layer. Thus, xylem regeneration would open the possibility of extending the cambium layer, which in turn, stimulated phloem development in wounded regions. Consequently, the formation of novel vascular tissue led to a robust root system with ARs directly connected to the sieve elements of the phloem (**Figure 2G**). In microcuttings of Rhododendron, a more robust vascular system and enhanced rooting were related to a larger phloem:xylem width ratio (Dokane et al., 2014). Moreover, a recent report by Wamhoff et al. (2024) mentioned that lignin presence in rose cuttings may represent a mechanical barrier to AR outgrowth and reduce rooting capacity in some genotypes. Therefore, the stimulating effect of the laser treatment might be related to the reduction of mechanical strength by eliminating sclerenchyma fibers, thus, allowing an increase of vascular tissue.

According to Damodaran and Strader (2024), cells close to the basal wound are competent for AR formation, whereas in distal cells this capacity is reduced. The results of our study show that in cuttings containing two wounds at the same time, AR formation is still preserved in the basal wound. Furthermore, the positioning of ARs seems to be related to the vascular connection between the leaf-bud and the basal wound. This phenomenon seems to be related to a higher physiological activity in this cutting section as evidenced by the synthesis of new xylem on the leaf-bud side in control cuttings (**Figure 2**), or the permanent higher levels of free IAA in the stem section below the axillary bud in all treatments (**Figure 6**). These results suggest that additional laser wounds may impact long-distance signaling, while the vascular connection between the leaf-bud and the basal wound is permanently more active, and therefore, a site with greater competence for AR formation. Laser-treated cuttings displayed an increase in the total number of roots per treatment and root fresh mass per rooted cutting. Moreover, additional analyses showed that ARs from laser-treated cuttings were longer and had more lateral roots (**Supplementary Figure S1**). These results correspond to those reported by Szajsner and Bąbelewski (2014) for laser treatment of *Viburnum rhytidophyllum* cuttings, and by Morales-Orellana et al. (2024) in leafy stem cuttings of rose under different laser pattern designs. Recent studies in wounded tissues of *Arabidopsis* mentioned that local water availability determined regeneration fates (Kareem et al., 2024). According to these authors, higher water availability regulated auxin response maxima which triggered *de novo* root formation, while low water availability gave rise to wound-induced callus, reduced spatial auxin response and inhibited root regeneration. Therefore, the stimulation of AR formation reported in our study may also be related to a higher local water availability through laser wounding.

### 4.2 Biochemical response

#### 4.2.1 Jasmonic acid and abscisic acid indicate a local stress response

The capacity of jasmonic acid (JA) to respond to stress triggered by tissue excision at the cutting base has been reported for petunia (Ahkami et al., 2009; Lischweski et al., 2015), carnation (Agulló-Antón et al., 2014) and pea (Rasmussen et al., 2015) cuttings. According to these reports, JA presents a rapid reaction during the first minutes after excision similar to our results. However, because the temporal design of our experiment involved not only the time needed for additional laser wounding, but also the time period since the cutting excision from the mother plant, more sampling time points during the first hours would be needed to get a clearer picture about the early dynamics of JA in response to the additional wounding and to compare the data to literature.

As expected, a typical characteristic of laser-treated cuttings was a higher concentration of JA in the laser-treated sections S_LS_ and S_OS_ compared to the same uninjured regions of the control. Although the reasons for this JA increase are directly related to greater wounded tissue, the type of exposed tissue also plays a crucial role in the JA levels. Woody cuttings of *Platycladus orientalis* contained significantly higher levels of JA in the phloem compared to the xylem within minutes after wounding (Liu et al., 2021). Thus, a particular reaction of phloem tissue due to the laser effects in its surrounding may also explain the high concentrations of JA directly in the laser-treated zones. The laser treatment resulted in a high concentration of JA-Ile in the laser-treated compared to cutting base sections. At first glance, it may seem that laser-wounded regions intensified the JA signal in the cutting base sections. However, sampling of the laser-wounded cuttings involved a longer time period after the first wounding and laser excision, which may have caused JA to accumulate. Thus, the interpretation of the data at 0 DAT should be done with care, as due to labor organization basal and laser wounds were applied at different time points, and the early reaction in JA and JA-Ile is known to be highly dynamic (Schulze et al., 2019).

JA biosynthesis is a crucial plant defense response, not only in locally damaged tissues, but it is also necessary to transport wound-induced signals to distal plant organs and thus to elicit a systemic response (Lee and Seo, 2022). During this process, N-jasmonoyl-L-isoleucine (JA-Ile), the active form of jasmonic acid, plays an important role in translocating signals between damaged and undamaged zones (Morin et al., 2023). Experiments conducted by Chauvin et al. (2013) demonstrated that JA-Ile synthesis occurs immediately after wounding and it accumulated in wounded tissue and healthy tissue vascularly connected. This phenomenon may partially explain why relative high levels of JA-Ile were also recorded in the leaf-bud section of control cuttings during 0 DAT (**Figure 4B**). The effect of JA modulation, its interaction with other plant hormones, and its relationship with AR formation are still under investigation. It appears that JA has a stimulating function in excision-induced AR formation, particularly during the induction phase as shown by pulse treatments of pea cuttings (Rasmussen et al., 2015) and is further reviewed in Druege et al. (2019).

The ABA concentration pattern at 0 DAT suggested that the presence of this phytohormone was also related to an early defense response after wounding. Similar to JA, the levels of ABA in laser-treated cuttings were higher compared to the control. In contrast to JA, ABA accumulation reached its maximum at 1 DAT, and it remained elevated in concentration until 4 DAT. Endogenous ABA levels in wild-type potato and tomato plants showed a similar reaction to wounding, where ABA concentration doubled within 24 h after wounding in wounded and non-wounded tissue, triggering a systemic induction response (Peña-Cortés et al., 1989). Da Costa et al. (2013) and Lakehal and Bellini (2019) considered ABA as inhibitory for AR formation. However, recent studies suggest that ABA may positively influence rooting in woody plant cuttings of some species. Vaičiukynė et al. (2019) observed a 6-fold higher accumulation of ABA in birch (*Betula pendula*) genotypes that were able to root, compared to recalcitrant genotypes. However, this response was not found in cuttings of *Populus* species in the same study. Recent studies by Qin et al. (2023) indicated that ABA induced the expression of auxin biosynthesis genes and auxin accumulation in roots. According to Emenecker and Strader (2020), ABA can promote or repress auxin activity depending on specific interactions. It is known that ABA is linked to water availability (Kareem et al., 2024) and plays a critical role in regulating root system architecture (Kou et al., 2022) and osmotic stress responses (Ma et al., 2019). Interestingly, in the basal wounded region ABA concentrations were significantly lower in both laser treatments compared to the control, specifically at 1 DAT (**Figure 5**). These results could point to a higher water availability or reduction in osmotic stress in the base of the laser-treated cuttings, and thus, a better condition for AR formation (Kareem et al., 2024). The relationship of ABA and AR formation needs further investigation, especially because additional analysis of its conjugated form, abscisic acid glucosyl ester (ABAGlc), and its oxidized product, phaseic acid (PA), showed complex dynamic patterns (**Supplementary Figure S3**).

##### 4.2.1.1 Laser wounded tissue contained more auxin amino conjugates and had different IAA-cytokinin ratios

The distribution of free auxins and auxin conjugates clearly demonstrated the conserved basipetal transport to the cutting base regardless of the treatment (**Figure 6**). In case of IAA, its increase in the cutting base after a rapid peak of JA has been reported in cuttings of petunia (Ahkami et al., 2009, 2013) and pea (Rasmussen et al., 2015). A similar dynamic of JA and IAA in leaf explants of *Arabidopsis*, and the finding that blocking of JA response inhibited rooting, whereas this effect could be rescued by auxin application, indicated that wound-induced JA may stimulate rooting via stimulation of auxin action (Zhang et al., 2019). However, despite the pronounced effect of laser wounding on JA levels and rooting (**Figure 4A**), IAA levels showed only a minor local response in the additional wounded, but not in the basal section, where rooting was mostly recorded.

Both laser treatments resulted in an increase in concentrations of the auxin amino acid conjugates IAAsp and IAGlu. It has been shown in cuttings of petunia, that soon after the rise of free IAA in the stem base (Ahkami et al., 2013), transcripts of GH3 proteins, which may function as IAA-amidosynthetases were upregulated (Druege et al., 2014). Yang et al. (2019) showed, that IAA apically applied to petunia cuttings is transported to the stem base but it is rapidly metabolized. Considering these findings, the increased concentrations of IAA conjugates and OxIAA reflect a stimulated conjugation and oxidation of free IAA in the tissues. Because the levels of free IAA were not reduced by the laser treatment, the results as a whole point towards a stimulated auxin influx into, or biosynthesis, in the tissues. This may have resulted in locally and/or temporarily elevated IAA levels in specific tissues or cells in response to the laser treatment, that were not detected in the present study. Although it is well known that potential auxin sources are located in the upper parts of cuttings like buds and developed leaves, recent studies indicate that additional sites of auxin production are in and around dead cells (Matosevich et al., 2022; Kacprzyk et al., 2022). This is because the natural auxin IAA can be derived from the tryptophan pathway, where this amino acid is released from proteins by hydrolytic enzymes when cells autolyze and die (Sheldrake, 2021). Interestingly, this supply of IAA also plays a crucial role in the maintenance and differentiation of vascular tissue, particularly xylem, which maintains cellular quiescence of organizer cells (Smetana et al., 2019). These statements led us to suggest that laser-treated regions became potential sites for generating IAA, which was conjugated to IAAsp and IAGlu. IAA-conjugates prevent an excess of free IAA in the tissue, and become a reserve of auxins to be hydrolyzed later depending on the plant’s demand (De Klerk et al., 1999). Our results also showed a higher and irreversible inactivation of IAA in form of OxIAA, especially in the bases of laser-treated cuttings (**Figure 6D**). Hayashi et al. (2021) observed a greater increase in IAAsp compared to IAGlu due to its faster conversion from IAA at high IAA concentration. In *Arabidopsis thaliana*, alterations in the local amino conjugation of auxins lead to changes in root elongation and root density (Guo et al., 2022). Moreover, an increase in IAAsp and IAGlu has been related to a higher stimulation in the number of lateral roots per primary root length in *Arabidopsis* by Aoi et al. (2020), which is very similar to the increase in number of lateral roots per AR and individual root length in our study (**Supplementary Figure S1**). Since auxins in higher concentrations inhibit the root growth in later phases of AR formation (De Klerk et al., 1995), its preferred conjugation and degradation in laser-treated cuttings after the induction phase may be beneficial for rooting. Consequently, the higher absolute number of ARs per treatment with greater root fresh mass (**Figure 3**) and longer roots with more lateral roots in laser-treated cuttings in our study could be driven either by stimulation of root induction by temporarily and locally higher IAA levels that caused secondary accumulation of IAA conjugates and OxIAA, or indirectly to the fast conjugation and degradation of IAA that stimulated root initiation.

IAA is the most important physiologically active native auxin. It is commonly accepted, that an accumulation of IAA in the cutting base is necessary during the root induction phase (Ahkami et al., 2013; Agulló-Antón et al., 2014; Rasmussen et al., 2015). De Klerk et al. (1995) showed that the induction phase of AR formation is promoted by auxins and a low level of cytokinins. In our study, cuttings at 0 DAT contained the lowest levels of cytokinins and highest levels of IAA in all treatments. Since auxin and cytokinins are considered antagonists during AR induction, differences in rooting response had been specifically related to changes in the auxin:cytokinin ratio in previous studies (da Costa et al., 2013; Otiende et al., 2021). Therefore, additional calculations of IAA:cytokinin ratios were carried out showing punctual significant changes particularly in the ratio IAA:tZR, IAA:cZR and IAA:sum of all cytokinins (**Supplementary Figure S5**). Interestingly, the laser treatment on leaf-bud side (L’LS) resulted in a significant increase in rooting percentage and also presented significant differences in the ratios IAA:cZR at 1 DAT and IAA:tZR at 2 DAT in several cuttings sections compared to the control. Therefore, the significantly lower ratio at day 1 and day 2 in the stem section of leaf-bud side wounded cuttings suggests that this lower ratio stimulated root induction. Although the present study did not include analysis of active trans-zeatin (tZ), the presence of trans-zeatin-riboside (tZR) in laser-wounded sections of the laser treatment on leaf-bud side (L’_LS_) indicated a higher fraction of this cytokinin being xylem-mobile, that can be enzymatically converted into physiologically active tZ. Finally, the increase in tZR in stem sections of leaf-bud side wounded cuttings coincides with that reported by Ikeuchi et al. (2017), where wounding resulted in the activation of cytokinin biosynthesis like tZRs and activation of multiple developmental regulators. Recent reports by Damodaran and Strader (2024) highlight the role of CK signaling in the generation of AR competence zones along the hypocotyl of *Arabidopsis*. Analyses of gene expression in several regions along the hypocotyl revealed that KMD2 as regulator of CK signaling was rate-limiting for the regulation of AR formation competence and able to influence auxin transporters (Damodaran and Strader, 2024). Since not only the concentrations of plant hormones matter, but also signaling is important, the expression of candidate genes involved in hormone perception and signaling as well as vascular formation-related genes as those identified by Matsuoka et al. (2021) should be analyzed in upcoming studies.

##### 4.2.1.2 Sucrose and starch were locally depleted in laser-treated sections

Laser-wounded tissue obviously became a sink for tissue repair, reconnection processes (including callus and xylem formation) and AR formation. Studies on carbohydrate dynamics of petunia cuttings during the first days of cultivation indicate that AR formation was associated with a rearrangement of metabolic pathways including local changes in carbohydrate-related enzyme activities from the beginning of cultivation (Ahkami et al., 2009). According to these authors, carbohydrate dynamics during the first days are characterized by a sink establishment phase starting at 0 DAT at the cutting base, which is characterized by temporal sugar depletion particularly of hexoses. Later, a recovery phase lasted around up to 3 days, and a ‘maintenance phase’, characterized by constant symplastic transport and strong increase of sugars translocated from source leaves into the stem base. Interestingly, the sink establishment and beginning of recovery phase were observed as a trend in the fluctuation of glucose and fructose in the cutting base section S_CB_ for both laser treatments and control in our experiments (**Supplementary Figure S4**). Ahkami et al. (2009) and Agulló-Antón et al. (2011) discussed a crosstalk between plant hormone pathways such as JA and IAA and sugar metabolism, particularly considering invertases. In petunia, it was shown that activity of invertases was dependent on functional polar auxin transport in the cuttings (Ahkami et al., 2013). Interestingly, in the same study blocking of polar auxin transport not only inhibited rooting but furthermore enhanced accumulation of sugars in the stem base as rooting zone. Therefore, the lower sugar levels in the stem base of the rooted cuttings were explained by the higher carbon utilization resulting from the root development.

Since the nodal cuttings had a single fully developed leaf, AR formation is considered as particularly dependent on this source organ for providing sugars and energy (Otiende et al., 2021). Despite this, our results show that starch reserves were not significantly different between the basal sections S_CB_ and laser-treated sections S_LS_ and S_OS_ (**Figure 8B**), even though both wound sites should compete for resources (Ma et al., 2023). The observed progressive decrease of starch in the laser-wounded sections S_LS_ and S_OS_ until 8 DAT suggest a disturbed carbohydrate transport influx from the leaf or higher carbohydrate utilization resulting from cell proliferation. Unlike starch, the distribution of sucrose in laser-treated cuttings depicts a tendency to be less present in the cuttings base section S_CB_ compared to laser-treated sections S_LS_ and S_OS_. Differences in sucrose concentration between wounded sections at 4 DAT and 8 DAT suggest the use of currently arriving assimilates for tissue repair (callus and vascular tissue growth) in laser-treated sections and possibly also for the increased rooting in basal section S_CB_. Interestingly, the content of glucose and fructose in the laser-treated sections show a trend to remain elevated at 8 DAT, similar to control cuttings on the leaf-bud side (**Supplementary Figure S4**). The relatively high concentration of both monosaccharides in these regions coincide with the increase in vascular growth for control and laser-treated cuttings (**Figure 2**). According to Ahkami et al. (2013), higher concentrations of glucose and fructose could be related to the invertases activity which have been shown to be activated by auxin in petunia. Significantly higher ratios of (glucose + fructose):sucrose at 8 DAT in laser-treated sections S_LS_ and S_OS_ suggest that invertase activity may have been enhanced by the additional wounding (**Supplementary Figure S4**). In conclusion, the carbohydrate data indicates a higher carbohydrate utilization in the wounded sections S_LS_ and S_OS_ in response to the additional laser wounding for the locally induced tissue repair, and by the stimulated rooting in the stem base section S_CB_. More time points are needed to gain a proper understanding of the carbohydrate dynamics and its cross-talk with plant hormones.

## 5 Conclusion

The present study demonstrates that laser treatments triggered a complex and dynamic biochemical reaction in the laser-treated regions and in uninjured regions during the first days of cultivation in rose cuttings. In general, it can be concluded that laser treatment and the resulting presence of additional laser-injured tissue increased the stress-sensitive plant hormones in the cuttings. JA increased only during the first hours, while the increase of abscisic acid was prolonged and not limited to wounded areas. Contrary to expectations, laser treatment did not alter the dynamics of the most important physiologically active auxin IAA at the investigated temporal and spatial level, but resulted in a considerable increase in auxin conjugates. A strong increase in IAA conjugates and the oxidation product OxIAA in the basal stem section in response to the additional wounding coincided with the improved root quantity and quality (absolute root number per planted cutting and root mass and root length per rooted cutting) and pointed indirectly to a high temporal or local abundance of IAA. In this context, laser-wounded tissue may have produced an ‘additional’ source of auxin compounds. Unlike auxins, cytokinins responded differently: A temporal and significant increase of tZR and cRZ corresponded to the laser treatment applied on the leaf-bud side, which also presented the highest rooting percentage. In consequence, the laser treatment significantly decreased the IAA:cytokinin ratio, which likely was more favourable for AR induction. Moreover, the decrease in sucrose and starch content in areas with tissue excision indicated that damaged areas became sink regions, i.e., zones that require resource consumption for healing and tissue reconnection in laser-treated sections, while in the basal section for the stimulated root development. Further studies with more early sampling time points are necessary to fully understand the functional relationships between laser-induced additional wounding of cuttings, plant hormones, primary metabolites and rooting. Precise laser treatment of cuttings appears as potential tool to improve propagation protocols in the future particularly for plants with low rooting capacity.

## Data Availability

The raw data supporting the conclusions of this article will be made available by the authors, without undue reservation.
