# Supplementary material for: Laser-wound stimulated adventitious root formation of Rosa canina cuttings involves a complex response at plant hormonal and metabolic level
Source: Front Plant Sci. 2024 Dec 16;15:1515990. doi: 10.3389/fpls.2024.1515990 (PMC11682910; doi:10.3389/fpls.2024.1515990)
Supplement: Supplementary file 1 [file DataSheet1.docx]

**Laser-wound Stimulated Adventitious Root Formation of *Rosa canina* Cuttings Involves a Complex Response at Plant Hormonal and Metabolic Level**

$Raul Javier Morales\mathrm{Orellan}a^{a,b}$, ${Thomas Rath}^{a}$,

${Uwe Druege}^{c},$ ${Yudelsy A. Tandrón}^{d}$, ${Nicolaus von Wirén}^{d},{Traud Winkelmann}^{b}$

${}^{a}{Hochschule Osnabrück}$ - University of Applied Sciences, Biosystem Engineering Laboratory (BLab), Oldenburger Landstraße 24, D-49090 Osnabrück, Germany

${}^{b}{Leibniz University Hannover}$, Institute of Horticultural Production Systems, Section Woody Plant and Propagation Physiology, Herrenhäuser Straße 2, D-30419 Hannover, Germany

${}^{c}{Erfurt Research Centre for Horticultural Crops,}$ University of Applied Sciences Erfurt, Kühnhäuser Str. 101, D-99090 Erfurt, Germany

${}^{d}{}$Leibniz Institute of Plant Genetics and Crop Plant Research, Department of Physiology and Cell Biology, Corrensstraße 3, D-06466 Gatersleben, Germany

***Correspondence:**traud.winkelmann@zier.uni-hannover.de

# Supplementary material

**Supplementary Methods S1** Extraction of plant hormones

The extraction of plant hormones started adding 1 ml ice-cold 50% aqueous methanol (v/v) containing the internal standards to each tube for phytohormone extraction. Samples were homogenized in a MM 301 vibration mill (Retsch GmbH, Haan, Germany) operating at the frequency of 27 Hz for 5 min and afterward sonicated for 3 min at 4°C using a Sonorex ultrasonic bath (BANDELIN electronic GmbH, Berlin, Germany). Samples were subsequently placed in the Reax 32 overhead shaker (Heidolph Instruments GmbH, Schwabach, Germany) for at least 30 min. After 10 min centrifugation at 14,000 rpm and 4°C (CT 15 RE centrifuge, Himac, Japan), the supernatant was transferred to clean safe-lock tubes. All samples were purified using Oasis PRIME HLB RP (1 cc per 30 mg), polymer-based Solid Phase Extraction (SPE) cartridges (Waters Co., Milford, USA). After loading the supernatant, the flow-through fraction was collected in a clean tube. The cartridge was then rinsed with 1 ml of 30% (v/v) ACN, and this fraction was collected in the same tube as the flow-through fraction. After this single-step SPE, the samples were evaporated to dryness at 40 °C in a vacuum concentrator RVC 2-33 IR (Martin Christ GmbH, Osterode am Harz, Germany) and stored at −20 °C until analysis.

**Supplementary Method S2** LC and MS/MS conditions for plant hormone measurements.

For LC, the used mobile phases were 0.1% FA. The flow was set to 0.4 ml min^-1^. The gradient conditions for auxin, abscisic acid and jasmonic acid analysis were as follows: A, 90% in 0 - 0.3 min; 90 - 80% in 0.3 - 0.7 min; 80 - 40% in 0.7 - 8 min; 40 - 1% in 8 – 8.5 min; 1% in 8.5 – 8.9 min; 1 - 90% in 8.9 – 9.0 min; 90% in 9.0 – 10.0 min. The gradient conditions for cytokinin analysis were as follows: A, 95% in 0 - 0.5 min; 95 - 90% in 0.5 - 1.0 min; 90 - 85% in 1.0 - 2 min; 85 - 1% in 2 – 8.0 min; 1% in 8.0 – 8.9 min; 1 - 95% in 8.9 – 9.0 min; 95% in 9.0 – 10.0 min. The Xevo TQ MS was operated in both, ESI+ and ESI- ion modes. The electrospray capillary voltage was 2.45 kV for auxins, abscisic acid, jasmonic acid, and cytokinins with a cone voltage of 20 V. The cone and desolvation gas flows were set to 20 and 1000 l h^-1^. For the quantification of the analytes we used three fragment ions, one for quantification and two for qualification. MS data were processed by using TargetLynx V4.1 SCN 904. The peak area of the diagnostic product ion was used for quantification. The limit of quantification was for cytokinins between 0.25 – 5, auxins 0.2 – 1.5, for abscisic acid 2.5 -5, for jasmonic acid 3.5 – 7 ng g-1 sample dry weight.


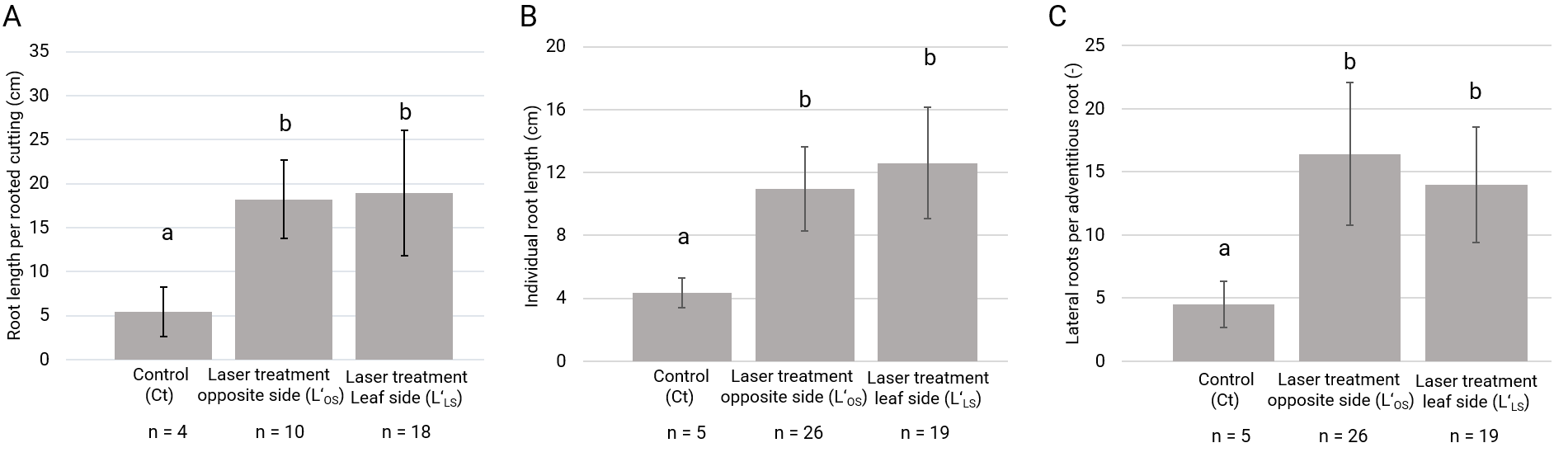


Fig. S1 Root length per rooted cutting (A), individual root length (B) and number of lateral roots per adventitious root (C). Different letters represent significantly different means between treatments (Tukey’s test, *P*≤0.05). Data are presented as mean values with standard error (SE).


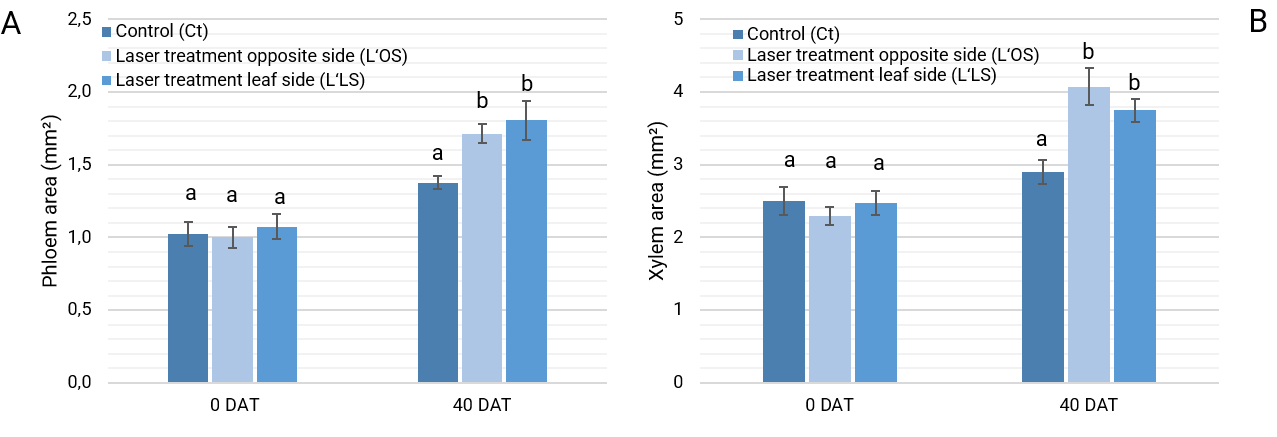


Fig. S2 Vascular tissue growth in terms of area for phloem (A) and xylem (B) at 0 DAT and 40 DAT. Different letters represent significantly different levels between treatments (Tukey test, *P*≤0.05). Mean values ± SD (n= 5 for 0 DAT, n= 4 for 40 DAT).


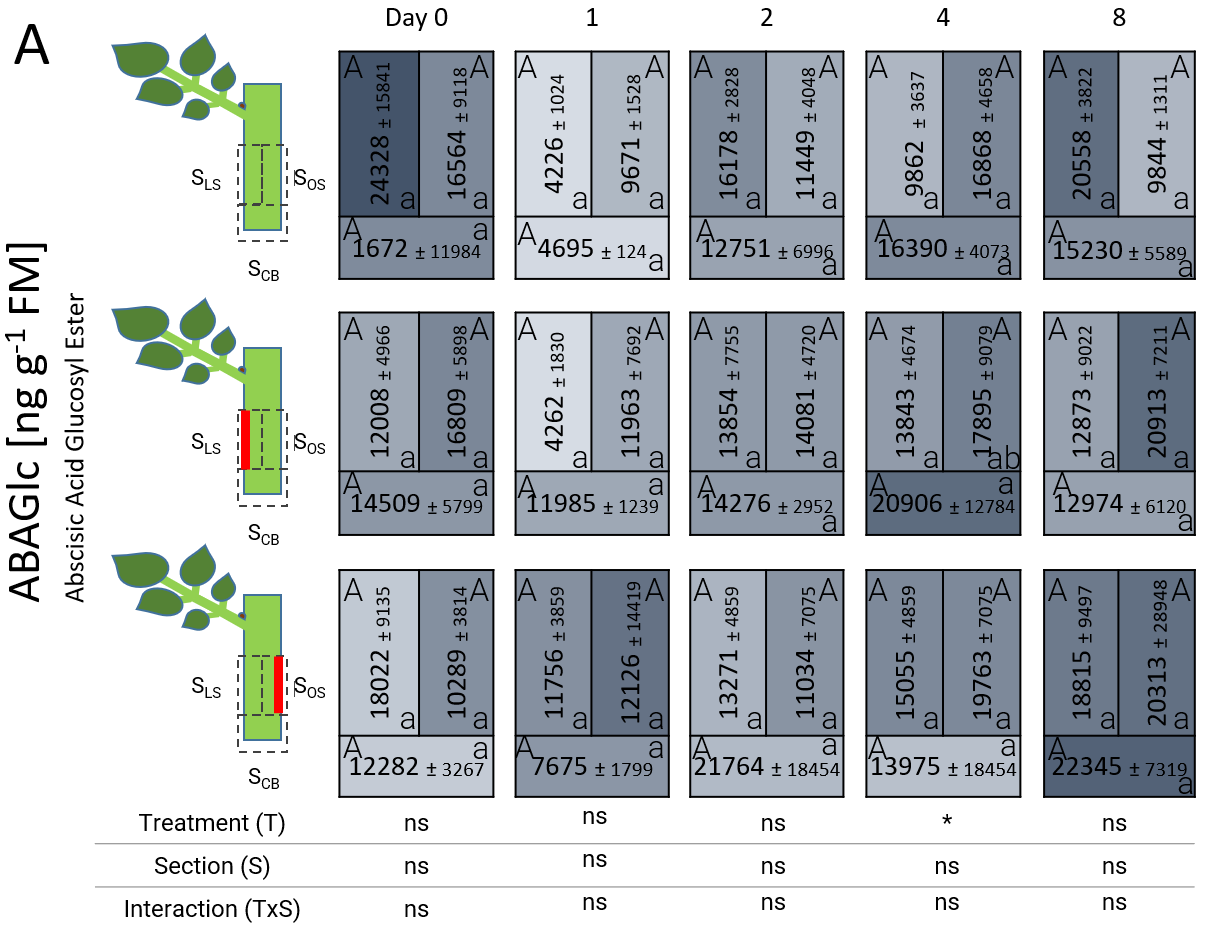


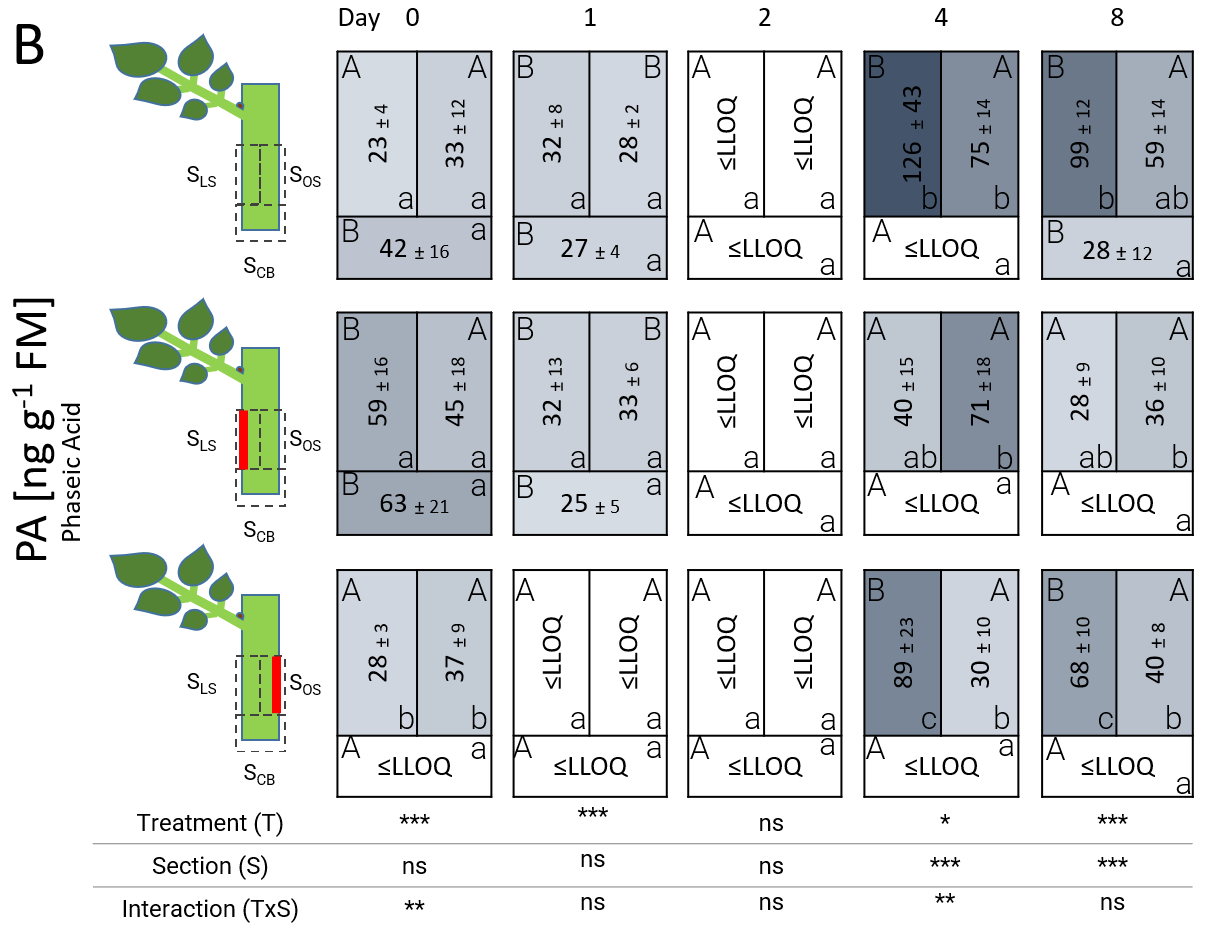


Fig. S3 Heat map depicting the distribution of abscisic acid glucosyl ester (A) y phaseic acid (B) analyzed over time and region for the different treatments represented as average + SD. The results of the ANOVA between the treatment factor and region with their interaction are shown at the end of each heat map. *, **, *** Significant at *P* ≤ 0.05, 0.01, 0.001 respectively; ns, non-significant. Capital letters represent significant differences between regions among treatments, while lowercase letters represent local significant differences between regions within each treatment (n=5, Tukey test, *P* ≤ 0.05). ≤LLOQ means Lowest Limit of Quantification.


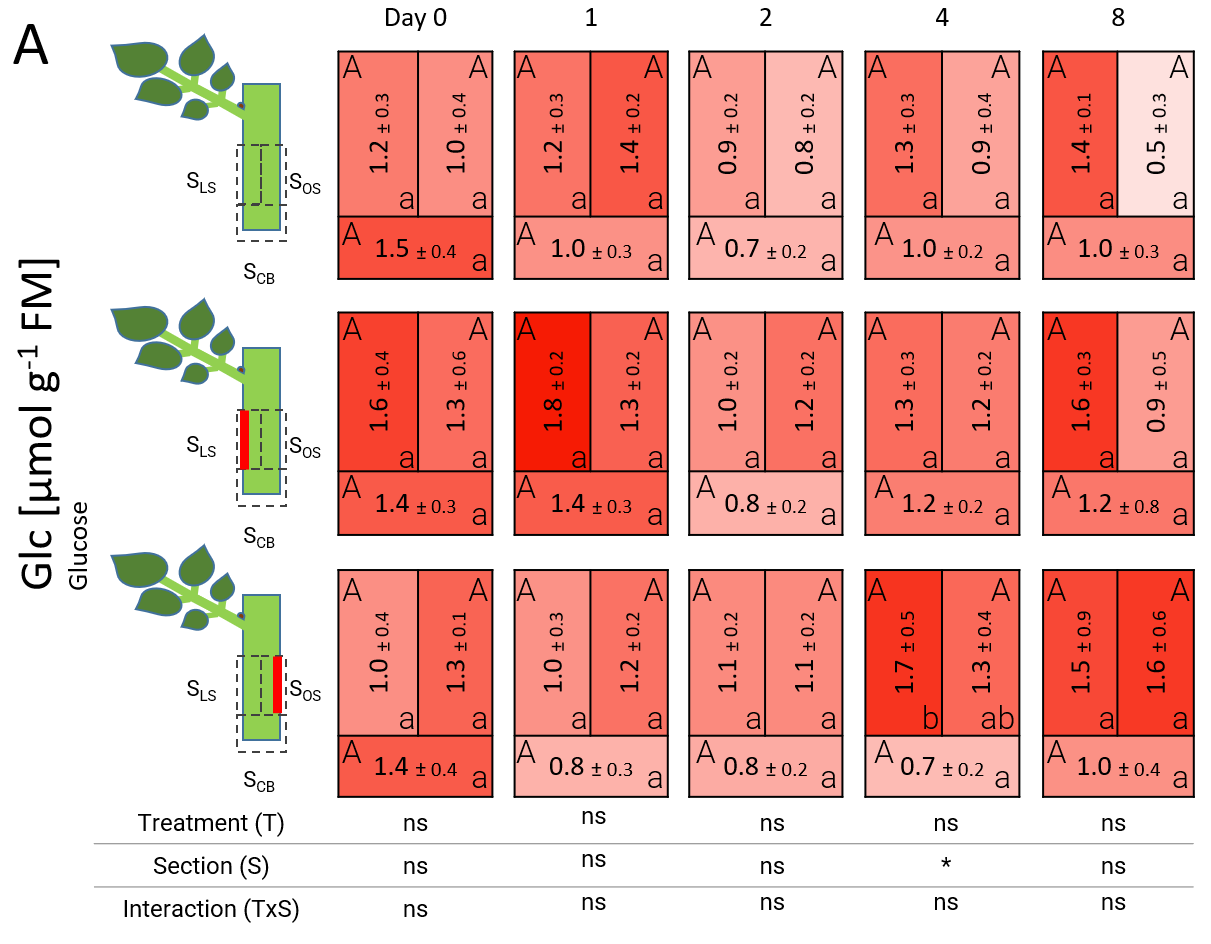


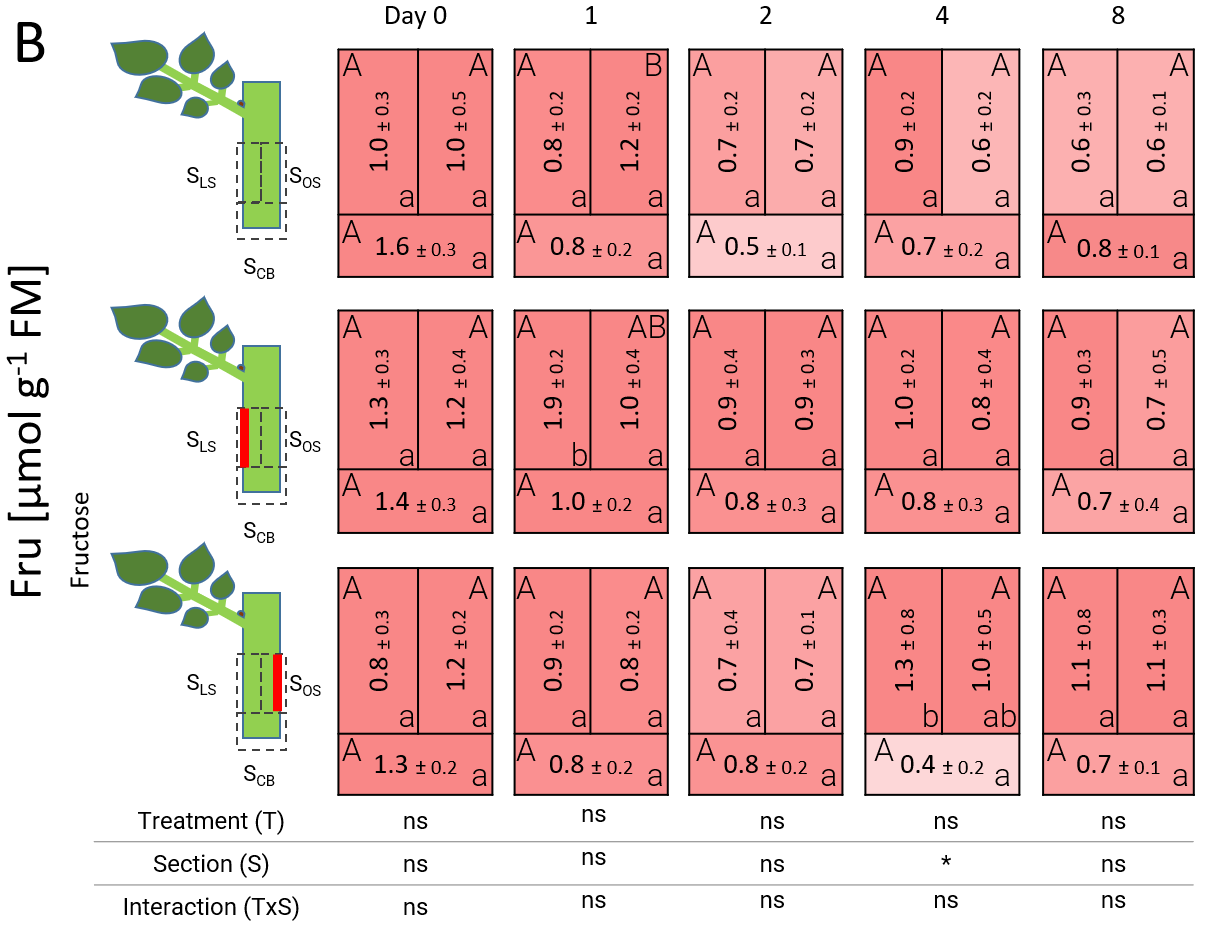


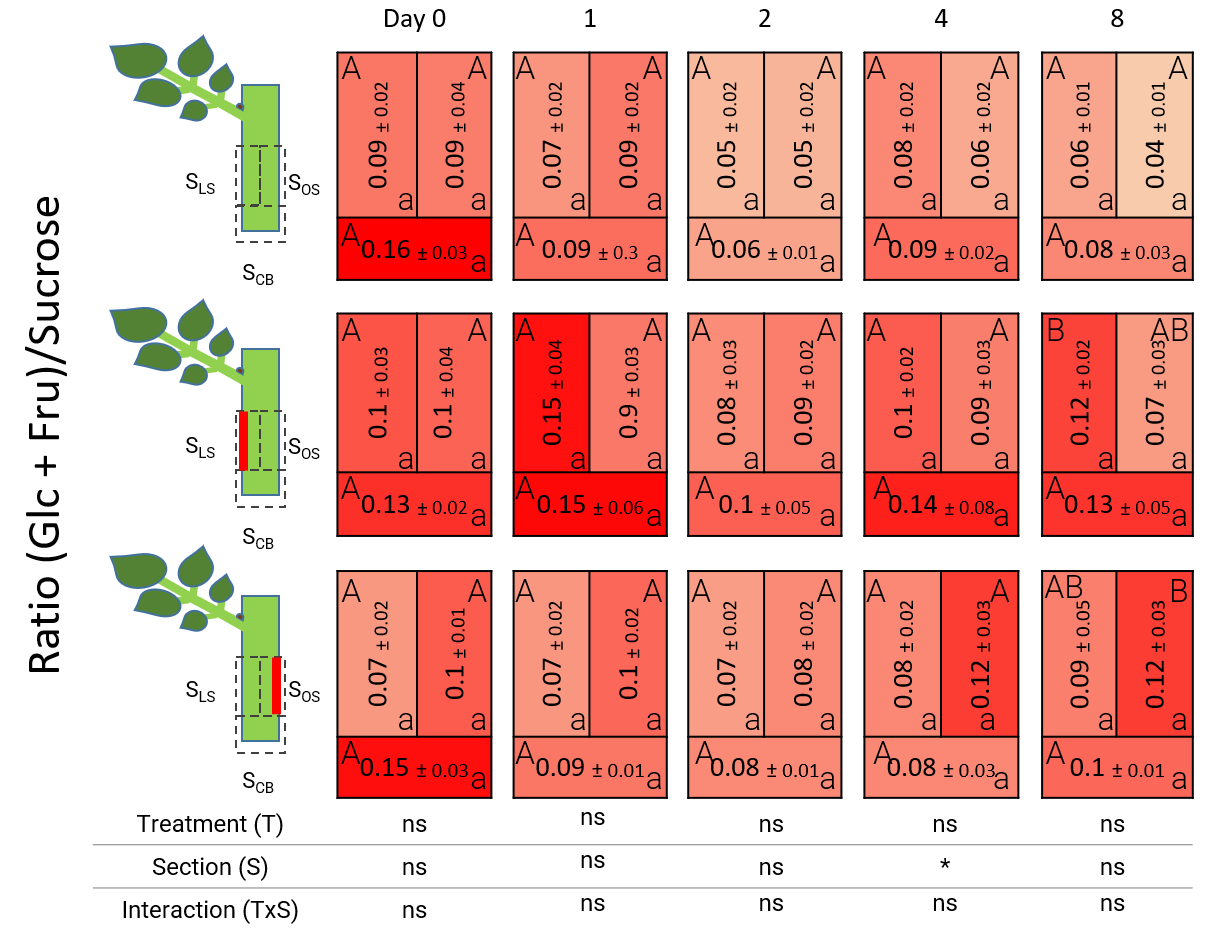


Fig. S4 Heat map depicting the distribution of glucose (A), fructose (B) and the ratio (glucose and fructose):sucrose (C) analyzed over time and region for the different treatments represented as average + SD. The results of the ANOVA between the treatment factor and region with their interaction are shown at the end of each heat map. *, **, *** Significant at *P* ≤ 0.05, 0.01, 0.001 respectively; ns, non-significant. Capital letters represent significant differences between regions among treatments, while lowercase letters represent local significant differences between regions within each treatment (n=5, Tukey test, *P* ≤ 0.05).


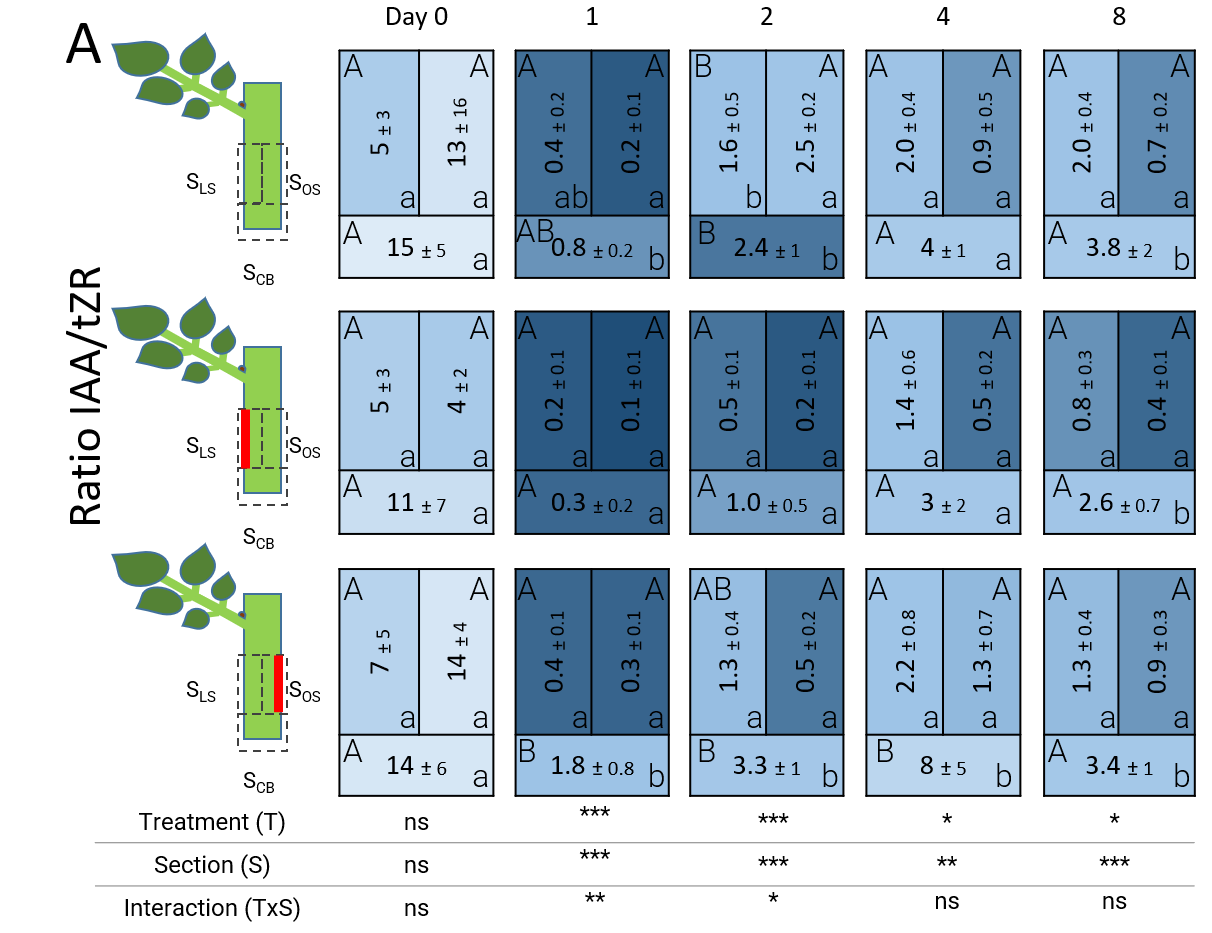


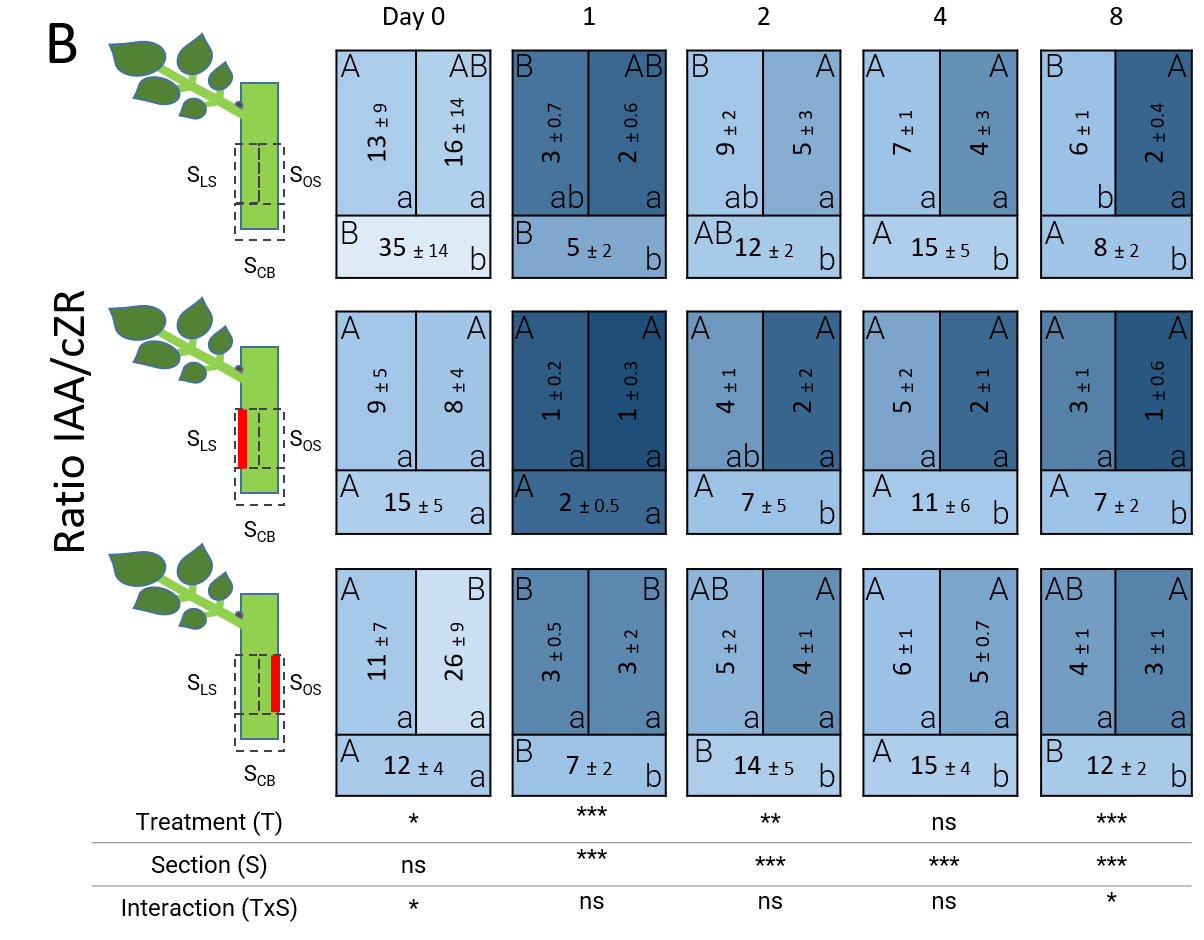


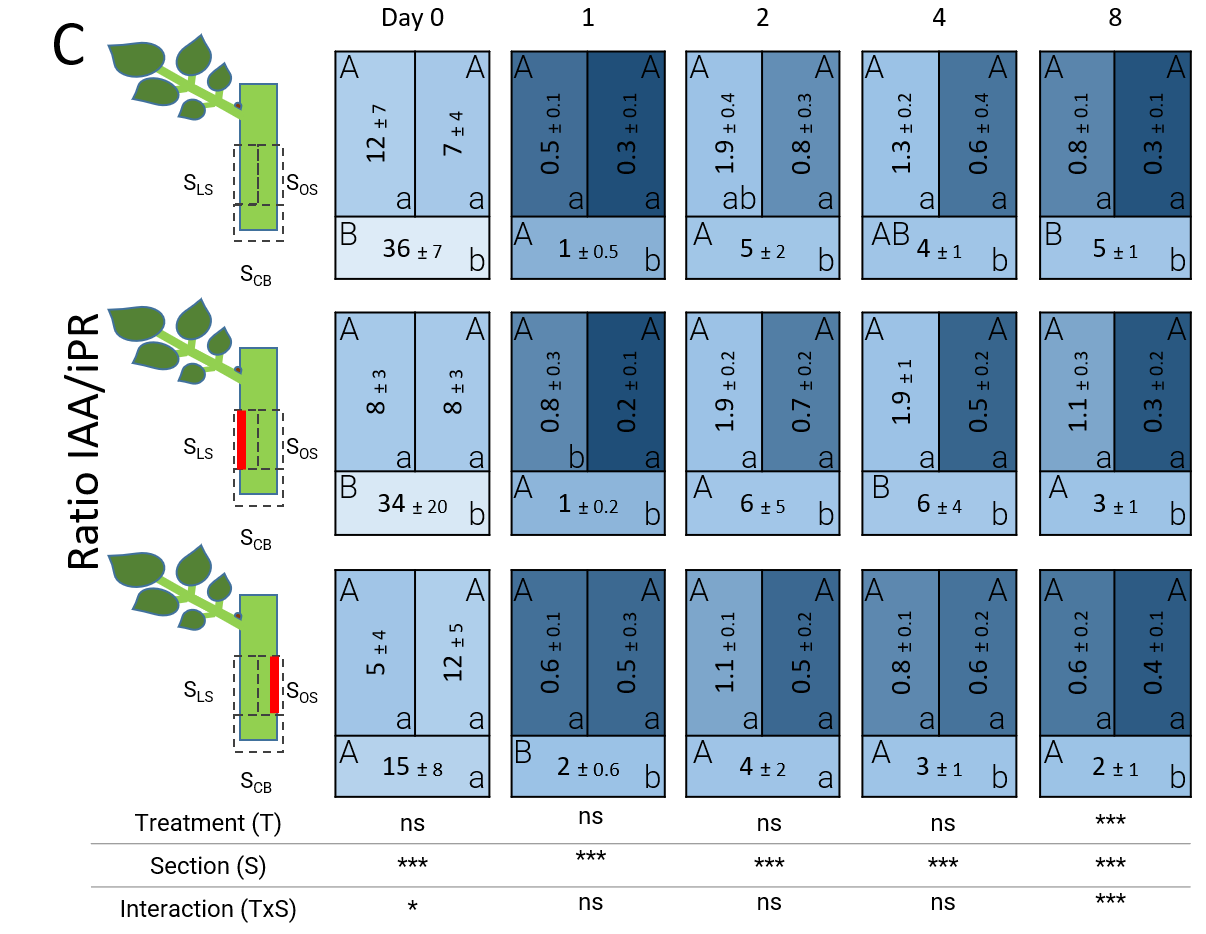


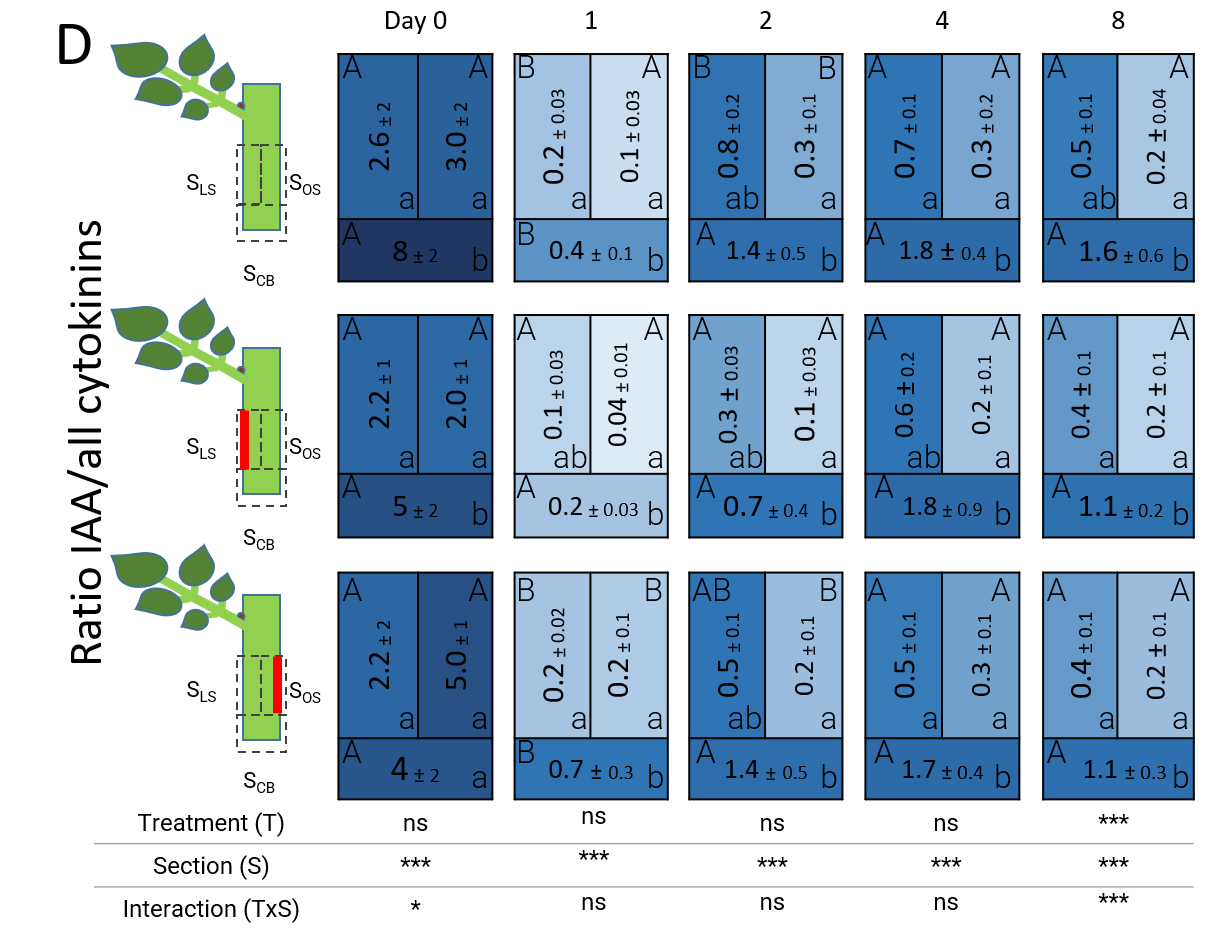


Fig. S5 Heat map depicting the distribution of IAA:tZR (A), IAA:cZR (B), IAA:iPR (C) and IAA:All cytokinins (D) analyzed over time and region for the different treatments represented as average+ SD. The results of the ANOVA between the treatment factor and region with their interaction are shown at the end of each heat map. *, **, *** Significant at *P* ≤ 0.05, 0.01, 0.001 respectively; ns, non-significant. Capital letters represent significant differences between regions among treatments, while lowercase letters represent local significant differences between regions within each treatment (n=5, Tukey test, *P* ≤ 0.05).
